# “LEARN”, a novel teaching method for Chinese clinical clerkship: A cross-sectional study

**DOI:** 10.3389/fsurg.2023.1113267

**Published:** 2023-02-13

**Authors:** Xiangyu Chen, Matthew F. Gong, Song Wu, Jinshen He

**Affiliations:** ^1^ Department of Orthopaedic Surgery, Central South University Third Xiangya Hospital, Changsha, China; ^2^ Department of Critical Care Medicine, Peking Union Medical College Hospital, Peking Union Medical College, Chinese Academy of Medical Sciences, Beijing, China; ^3^ Department of Orthopaedic Surgery, University of Pittsburgh Medical Center, Pittsburgh, PA, United States

**Keywords:** medical education—clinical skills training, clinical skills, undergraduate (MeSH), clerkship, cross-sectional study

## Abstract

**Background:**

Despite the clerkship being crucial in the training of a future doctor, no widely accepted education model has been proposed. This study devised a new model for clinical clerkship rotations, titled “LEARN” for Lecture, English-video, Advisor, Real-case and Notion, and evaluated whether the LEARN model is appropriate for medical education in China.

**Methods:**

A cross-sectional study was performed among 101 fourth-year students from the Xiangya School of Medicine during an Orthopaedic Surgery clerkship rotation in the Third Xiangya Hospital. They were divided into seven groups and took clerkship based on the LEARN model. A questionnaire was collected at the conclusion to measure learning outcomes.

**Results:**

The LEARN model was highly accepted with the acceptance of five sessions being 95.92% (94/98), 93.88% (92/98), 96.98% (97/98), 100% (98/98) and 96.94% (95/98). The outcomes of two genders were comparable, whereas a difference was observed in the test score among groups (group 3 scored 93.93 ± 5.20, higher than others). Quantitative analysis showed that positive correlations existed in participation in the Notion (Notion means students’ case discussion) section with leadership (*r* = 0.84, 95% CI: 0.72–0.94, *p* < 0.001), participation in the Real-case section with leadership (*r* = 0.66, 95% CI: 0.50–0.80, *p* < 0.001), participation in the Real-case section with mastery of inquiring skills (*r* = 0.57, 95% CI: 0.40–0.71, *p* < 0.001) and participation in the Notion section with mastery of physical examination skills (*r* = 0.56, 95% CI: 0.40–0.69, *p* < 0.001). Further qualitative analysis demonstrated that high-level participation in the English-video section indicated better outcomes in mastery of inquiring (*p* < 0.01), physical examination (*p* < 0.001), film reading (*p* < 0.01) and clinical reasoning (*p* < 0.01) skills.

**Conclusion:**

Our results support the LEARN model is a promising method for medical clerkship in China. Further research involving more participants and more meticulous design is planned to test its efficacy. For refinement, educators may try to promote students’ participation in the English-video session.

## Introduction

The transition period from the classroom to the clinic has long been regarded as a critical stage of medical education. Medical students face a series of challenges during this transition, including applying concentrated knowledge quickly in daily clinical practice, navigating an incompatibility between undertrained skills and high expectations of internship mentors, and learning how to communicate effectively with patients in real time (1, 2).

To make medical students transition smoothly, various education models have been taken, including pre-clerkship (3), peer-team (2), self-monitored e-learning (4–6), integrated smartphone apps (7) and mentoring programs led by residency directors (8). The most widely implemented transition model implemented in China is a clerkship rotation. Specifically, a clerkship is a stage when medical students enter the clinic and learn basic bedside skills by observation (9), instead of actual practice which usually begins during the fourth year of the 5-year clinical medicine program. Moreover, in consideration of the intense relationship between doctors and patients in China (10, 11), the clerkship period, which exposes medical students to the clinical environment, equips them with essential practical skills and provides students with opportunities to communicate with patients (12), certainly brings growth for future doctors in both their professionalism and communication skills. For Chinese medical students facing the crossroad between Master of Medicine and Master of Science degrees for further medical education (13), the experience of the clinical work and environment during clerkship boosts students to make decisions.

For clerkship, medical educators have been probing novel educational models: establishing a highly interactive environment (14), involving patients in undergraduate education (15), developing small groups among students (16), and implementing simulation programs (17, 18). Hybrid education methods have also been implemented for some subjects (19–23). No benchmark model for clerkship has been proposed previously, and with limited literature discussing this topic, there is a need for further research into effective teaching models.

This study proposes a novel model for medical education in clerkship—LEARN (Lecture, English-video, Advisor, Real-case and Notion), which consists of traditional lecture-based classes, English videos in high quality for Chinese students, directions from the advisor, real cases from hospital and notion discussed among students. Bilingual education was stressed in this model for several reasons. Firstly, since English is the universally used language in medical community worldwide, mastery of English enables a doctor to learn the cutting-edge medical knowledge in the first place and publish scientific work on international journals (24). Early orientation will improve undergraduates’ ability in medical academic English, which benefit them in their future career. Secondly, English teaching materials online in English are in better quality. This series of lessons not only exposed students to English environment, but also introduced English websites for medical education to them. We hope that students with good competence will find more intriguing videos from those websites, which might assist their learning. Thirdly, English level is indispensable assessing criteria for college students, as passing College English Test Band 4, also known as CET4, is required for graduation. The LEARN program exposed students to medical English, which may improve their English ability.

This single-institution, cross-sectional study aimed to verify the efficacy of a new modality of the clerkship class. The purpose of our study was to evaluate whether the LEARN model is appropriate for medical education in China.

## Methods

### Participants

This study was performed from April 2021 to June 2021. Participants were students who majored in clinical medicine and took the clerkship rotation in the hospital. The ethics committee of the Hospital approved the research (ID: 2021-S078).

### Study design

**Figure 1:**
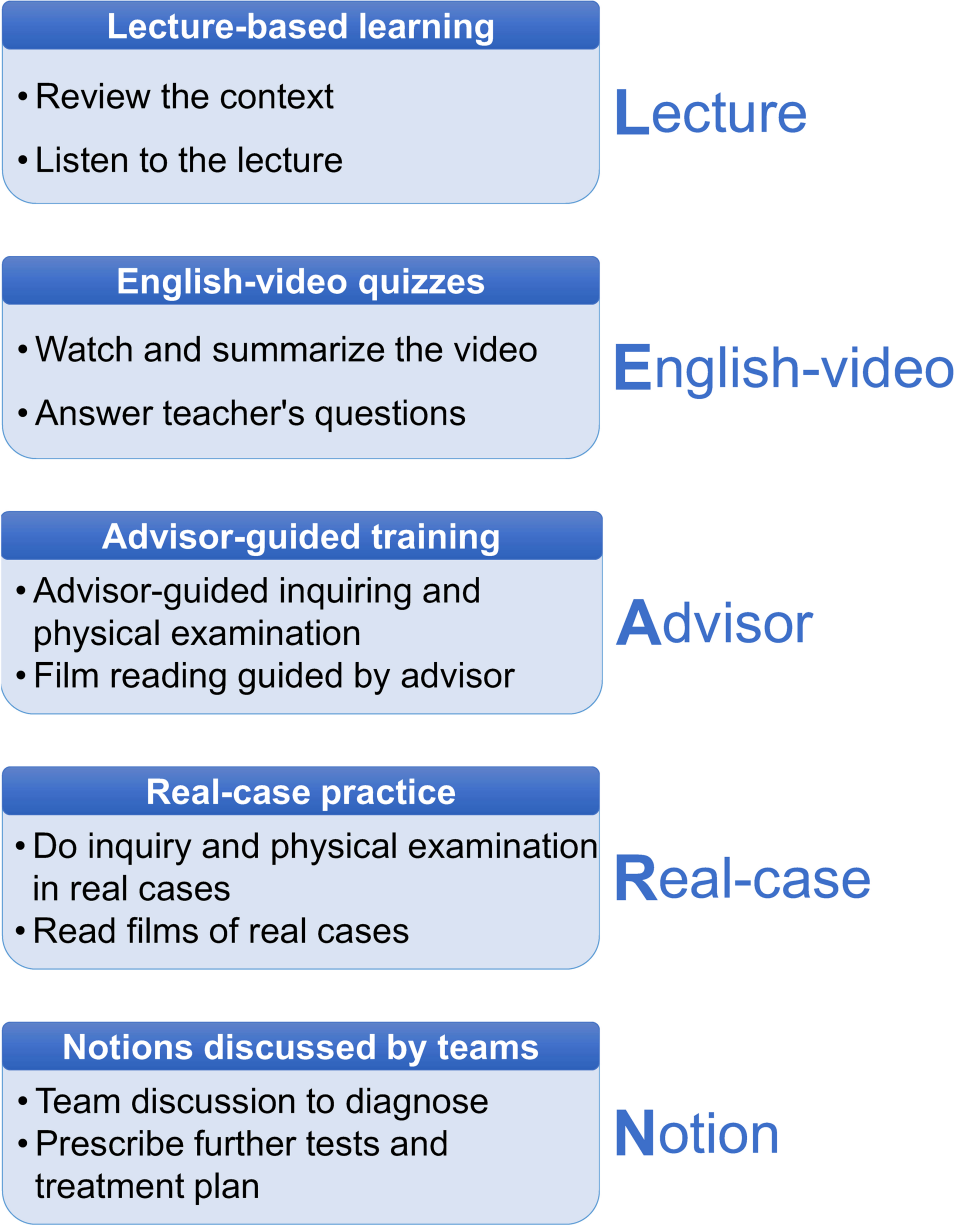
The process of LEARN model.

The LEARN model was implemented in three Orthopaedic Surgery clerkship rotations: (1) Arthroplasty and Orthopaedic Oncology; (2) Upper Extremity; and (3) Lower Extremity. The senior orthopaedic advisor (JH) lead each of the clerkship classes. Figure 1 demonstrates the design of the teaching activities. Students took clerkship classes in groups of 14–17 members. The advisor began by giving a lecture to review relevant content. Subsequently, informative English videos from YouTube (originating from Osmosis and MedicalScribe), introducing mechanisms of injury, clinical presentation, and diagnostic work-up, were displayed. At the end of the clip, students were required to answer questions related to this content. Afterwards, additional clinical skills were taught in various approaches with the advisor's assistance: the advisor emphasized key learning points using the Mind-Map feature on PowerPoints, conducted physical examination demonstrations, and teach radiographic film reading with clinical cases. After this training, groups of 3–4 students were formed to perform a bedside history and examination on selected patients. Every group was further questioned to discuss their diagnostic approach, intended work-up and provide management plans, which the advisor provided feedback on. Team-based discussion was encouraged. Finally, students took a post-course exam to measure their learning outcomes. Involved teaching materials was PowerPoints, example of which can be found in the supplementary files.

Additionally, we carried out an online questionnaire to collect feedback on student ratings on the class quality, participation in different sections, and self-rating on skill mastery following the course (Supplementary File 1).

### Data collection

The research collected three aspects of information: individual information, clerkship test scores and clerkship class ratings. Individual information included sex, group number, and age. A 5-point Likert scale model was employed for quantitative variables. Student ratings of five sections of LEARN included: (a) the usefulness of the lecture in their academic learning (letter L); (b) the usefulness of English video based learning (letter E); (c) the influence of advisor-provided feedback (letter A); (d) their self-rating of clinical skills acquired from real cases (letter R), which were further separated into four sections: history-taking, physical examination, radiographic film reading, and diagnostic work-up; and (e) students’ participation in the notion exchanging session or the group-based discussion (letter N). Students reported their participation level in the last four sessions (English-video, Advisor, Real-case and Notion), since the first part, Lecture, was regarded as a passive way of learning with the same level of participation by default. Students were asked to self-rate their mastery of knowledge, history, physical examination, film reading, and clinical reasoning ability. The level of leadership in the class was evaluated.

Furthermore, questionnaires evaluated the following ordinal categorical variables: (a) the appraisal of the advisor's feedback; (b) students’ willingness to being specific patients; and (c) the necessity of the surgery video. For qualitative analysis, statistics were dichotomized into yes/high (score 5 and 4 on a 5-point Likert scale) and no/low (score 3, 2, and 1).

### Statistical analysis

We employed SPSS V26.0 (IBM Corp., Armonk, NY, USA) for statistical analysis. Primarily, data of subgroups in an abnormal distribution were respectively processed, with sex-based subgroups analyzed with the Mann-Whitney *U* test, and group-based subgroups analyzed with the Kruskal-Wallis *H* test. To illuminate the correlation among factors, we conducted Spearman analysis on quantitative analysis. Statistically significant variables were further dichotomized and analyzed *via* the chi-square test.

## Results

### General information

The total number of feedback reports was 101, of which three were excluded for incomplete information. Among the 98 valid respondents from seven clerkship groups, 50 male and 48 female students were 20–22 years old (21.7 ± 1.3).

### Students’ rating of the five sessions

**Figure 2:**
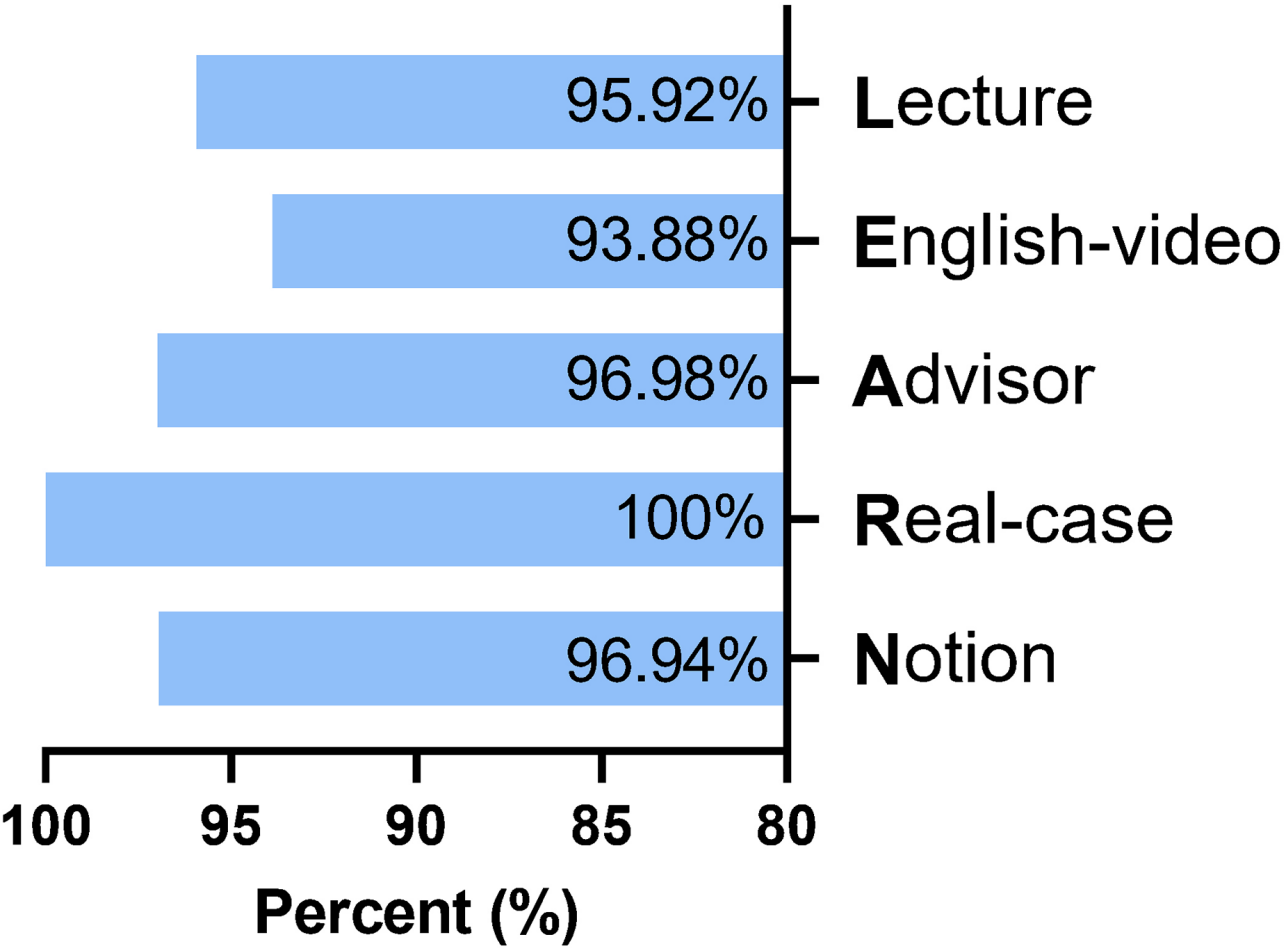
Students’ rating of the five sessions.

As Figure 2 showed, the Lecture session scored 4.80 ± 0.80 out of the 5-point scale coupled with 94 (95.92%) pieces of positive feedback, in which participants handed in a score higher than three points for the variable. For the English-video component, we observed a rating of 4.78 ± 0.41 and 92 (93.88%) pieces of positive feedback. For the Advisor component, the average score was 4.86 ± 0.34 with 97 students (98.98%) finding advisor-provided feedback promotive and beneficial. Quantitative outcomes of the Real-case component were 4.87 ± 0.34 and 98 (100%) pieces of positive feedback. The statistics of the Notion component showed an average grade of 4.81 ± 0.36 coupled with 95 (96.94%) pieces of positive feedback.

### Students’ opinions on clerkships based on the LEARN model

Our study also investigated other aspects of how students responded to this novel education model.

During the English-video session, 50 students (51.02%) admitted paying most of their attention to the animation, 18 students (18.37%) to the voiceover, and 30 (30.61%) to the vocabulary (Supplementary Table S1).

A large proportion of students were supportive of the physical examination demonstration, with 15 participants (15.31%) acting as a standardized patient (SP) in the class, 70 (71.43%) volunteering to be an SP, 6 (6.12%) noting reluctance in volunteering and 7 (7.14%) choosing not to answer (Supplementary Table S2).

The research also collected students’ reactions to the extensive videos provided on relevant Orthopaedic Surgery content at the end of the class. Over 90% of participants (89 students, 90.82%) recognized the utility of these videos for further providing clinical insights to integrate theory and practice (Supplementary Table S3).

For English-based instructional videos, all students were queried on what they thought the appropriate number of post-video quizzes was per students. Near 93% of students preferred the amount to be no more than three (Supplementary Table S4).

### Subgroup analysis

Table 1 demonstrates detailed information of feedback from male and female participants. There was significant variance in participation of Real-cases between male and female students, as females were more inclined to participate in the clinical practical skill learning session (4.72 ± 0.50 vs. 4.92 ± 0.28, *p* < 0.05). No other statistically significant difference between males and females was observed.

**Table 1:** Subgroup feedback of the clerkship class based on sex.

	Male	Female	Total
**Rating**			
Lecture	4.81 ± 0.79	4.79 ± 0.82	4.80 ± 0.80
English-video	4.72 ± 0.46	4.84 ± 0.33	4.78 ± 0.41
Advisor	4.82 ± 0.37	4.91 ± 0.29	4.86 ± 0.34
Real-case	4.82 ± 0.39	4.92 ± 0.28	4.87 ± 0.34
Notion	4.83 ± 0.35	4.80 ± 0.38	4.81 ± 0.36
**Participation**			
English-video	3.79 ± 0.71	3.94 ± 0.78	3.86 ± 0.75
Advisor	4.72 ± 0.90	4.92 ± 0.40	4.82 ± 0.71
Real-case	4.72 ± 0.50*	4.92 ± 0.28*	4.82 ± 0.41
Notion	4.68 ± 0.51	4.71 ± 0.51	4.70 ± 0.51
**Mastery**			
Knowledge	4.44 ± 0.70	4.50 ± 0.68	4.47 ± 0.69
Inquiring	4.42 ± 0.76	4.60 ± 0.61	4.51 ± 0.69
Physical examination	3.98 ± 0.98	4.27 ± 0.82	4.12 ± 0.91
Film reading	4.30 ± 0.84	4.46 ± 0.80	4.38 ± 0.82
Clinical reasoning	4.04 ± 0.90	4.35 ± 0.79	4.20 ± 0.86
**Leadership**			
	4.44 ± 0.86	4.60 ± 0.68	4.52 ± 0.78
**Score**			
	85.72 ± 7.34	86.44 ± 5.85	86.07 ± 6.63

*p < 0.05 between males and females.

As for group-based analysis, only the score of the after-class exam displayed a significant discrepancy among groups, with the average being 86.07 ± 6.73 (Table 2). Furthermore, comparison in pairs indicated that the average score of group 3 (93.93 ± 5.20) was higher than other groups, with group 1 being 82.23 ± 3.63 (*p* < 0.001), group 2 being 84.38 ± 4.19 (*p* < 0.01), group 4 being 82.50 ± 8.70 (*p* < 0.01), group 5 being 85.50 ± 8.45 (*p* < 0.01) and group 6 being 85.56 ± 4.76 (*p* < 0.01).

**Table 2:** Subgroup feedback of the clerkship class based on teaching group.

Group	1	2	3	4	5	6	7	Total
**Rating**								
Lecture	4.67 ± 1.11	5.00 ± 0.00	4.87 ± 0.30	4.61 ± 1.15	5.00 ± 0.00	5.00 ± 0.00	4.44 ± 1.40	4.80 ± 0.80
English-video	4.74 ± 0.34	4.67 ± 0.56	4.78 ± 0.41	4.83 ± 0.39	4.62 ± 0.52	4.90 ± 0.29	4.89 ± 0.27	4.78 ± 0.41
Advisor	4.88 ± 0.30	4.85 ± 0.38	4.6 ± 0.54	5.00 ± 0.00	4.89 ± 0.29	4.91 ± 0.27	4.93 ± 0.18	4.86 ± 0.34
Real-case	4.92 ± 0.28	4.92 ± 0.28	4.6 ± 0.51	5.00 ± 0.00	4.86 ± 0.36	4.94 ± 0.25	4.87 ± 0.35	4.87 ± 0.34
Notion	4.71 ± 0.41	4.85 ± 0.38	4.67 ± 0.44	4.92 ± 0.29	4.84 ± 0.30	4.84 ± 0.39	4.88 ± 0.33	4.81 ± 0.36
**Participation**								
English-video	4.12 ± 0.62	3.73 ± 0.86	3.87 ± 0.72	4.13 ± 0.83	3.86 ± 0.77	3.84 ± 0.68	3.57 ± 0.75	3.86 ± 0.75
Advisor	4.69 ± 1.11	4.69 ± 0.75	5.00 ± 0.00	4.83 ± 0.58	4.57 ± 1.12	5.00 ± 0.00	4.87 ± 0.52	4.82 ± 0.71
Real-case	4.85 ± 0.38	4.85 ± 0.38	4.67 ± 0.49	4.83 ± 0.58	5.00 ± 0.00	4.81 ± 0.40	4.73 ± 0.46	4.82 ± 0.41
Notion	4.46 ± 0.66	4.85 ± 0.38	4.60 ± 0.54	4.96 ± 0.14	4.75 ± 0.43	4.69 ± 0.57	4.60 ± 0.57	4.69 ± 0.51
**Mastery**								
Knowledge	4.46 ± 0.78	4.46 ± 0.66	4.20 ± 0.77	4.42 ± 0.79	4.71 ± 0.61	4.44 ± 0.73	4.60 ± 0.51	4.47 ± 0.69
History-taking	4.62 ± 0.65	4.46 ± 0.66	4.20 ± 0.77	4.58 ± 0.79	4.86 ± 0.36	4.38 ± 0.81	4.53 ± 0.64	4.51 ± 0.69
Physical examination	4.08 ± 0.86	4.00 ± 0.82	3.80 ± 0.86	4.42 ± 0.79	4.36 ± 0.74	4.13 ± 1.15	4.13 ± 1.06	4.12 ± 0.91
Film reading	4.62 ± 0.65	4.31 ± 0.85	3.93 ± 0.96	4.50 ± 0.80	4.50 ± 0.76	4.44 ± 0.81	4.40 ± 0.83	4.38 ± 0.82
Clinical reasoning	4.31 ± 0.85	4.08 ± 0.86	3.73 ± 0.80	4.42 ± 0.79	4.29 ± 0.91	4.38 ± 0.81	4.20 ± 0.94	4.19 ± 0.85
**Leadership**								
	4.46 ± 0.66	4.62 ± 0.77	4.40 ± 0.74	4.42 ± 1.08	4.64 ± 0.63	4.63 ± 0.72	4.47 ± 0.92	4.52 ± 0.78
**Score**								
	82.23 ± 3.63***	84.38 ± 4.19**	93.93 ± 5.20^a^	82.50 ± 8.70**	85.50 ± 8.45**	85.56 ± 4.76**	86.93 ± 2.46	86.07 ± 6.63

^a^ Significant difference only existed between group 3 and others.

**p < 0.01.

***p < 0.001 compared with group 3.

### Quantitative analysis

Spearman test was conducted among sixteen factors, which included nine independent variables, namely rating of five components (Lecture, English-video, Advisor, Real-case and Notion) and participation of four components (English-video, Advisor, Real-case and Notion), and seven dependent variables, including mastery of five skills (knowledge, history, physical examination, film reading and clinical reasoning), level of leadership, and score.

**Figure 3:**
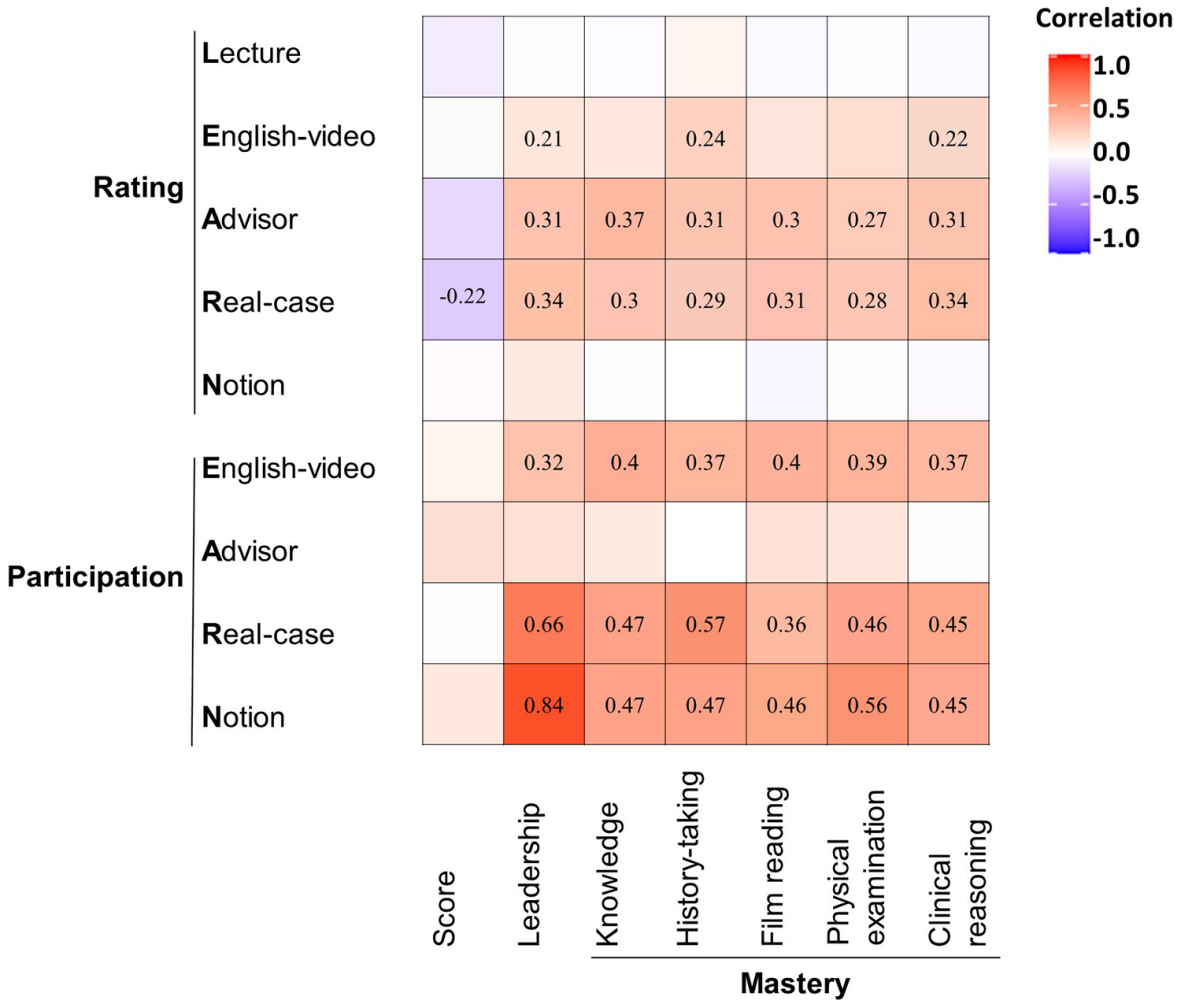
Corheatmap of variables involved in LEARN model.

Figure 3 displays the correlation between these variables. Correlation coefficient between participation of Notion and leadership was more than 0.8 (*r* = 0.84, 95% CI: 0.72–0.94, *p* < 0.001). Three pairs correlation coefficient were over 0.5: (1) participation of Real-case and leadership (*r* = 0.66, 95% CI: 0.50–0.80, *p* < 0.001); (2) participation of Real-case and mastery of history-taking (*r* = 0.57, 95% CI: 0.40–0.71, *p* < 0.001); (3) participation of Notion and mastery of the physical examination (*r* = 0.56, 95% CI: 0.40–0.69, *p* < 0.001). Additionally, there were nine pairs testified with correlation coefficient between 0.4 and 0.5, while twenty pairs were less than 0.4.

### Qualitative analysis

To further test for correlation, quantitative data was transformed into qualitative form, and the chi-square test was implemented in the form of a fourfold table (Supplementary Figure S1). Five pairs of variables were found with significant differences (*p* < 0.01): (1) participation in English-video and mastery of history-taking (*p* < 0.01); (2) participation of English-video and mastery of physical examination (*p* < 0.001); (3) participation of English-video and mastery of film reading (*p* < 0.01); (4) participation of English-video and mastery of clinical reasoning (*p* < 0.01); (5) participation of Notion and leadership (*p* < 0.01).

## Discussion

The novel five-component LEARN education model implemented in the clinical clerkship setting was considered highly effective by participating students. Keeping in line with our previous hypothesis, this new teaching model enhanced medical students’ comprehension of orthopaedics and prompted active learning. Additionally, with the assistance of electronic media, students were exposed to more comprehensive and cutting-edge educational resources that a traditional PowerPoint-based lecture could not provide (25, 26). Moreover, through integration with a clinical case, students experienced the simulated process of a clinical case from initial hospitalization to surgery, which exposed them to their role in future internships and possibly sparked their passion for pursuing a career as a surgeon (27).

Subgroup analysis indicated that both males and females benefited from the clerkship modality with similar after-class academic performance, though female participants reported a higher participation level in the Real-case session. Interestingly, there was statistically significant difference in post-test scores among seven clerkship groups. Group 3 demonstrated a much higher average score, 93.93 ± 5.20, compared to other groups. This phenomenon became intriguing since the participation outcomes of other groups seemed to outperform group 3, whose score excelled its counterparts, despite no valid significance. This outcome appeared to oppose the widely accepted conception that academic performance correlates positively with participation. Considering that students were randomly divided into seven clerkship groups, the possibility could be that a specific atmosphere, emphasizing independent theoretical learning and neglecting course participation, developed in group 3. Nevertheless, this hypothesis could not be further proved without more information on the students’ academic performance.

The Spearman test on quantitative variables hinted at directions for future improvement. Students’ ratings of Advisor and Real-case sessions were positively associated with their academic performance, practical skills mastery, as well as their leadership level, which indicated that emphasis should be placed on the two sessions mentioned above to advance the educational efficacy of the LEARN model. As for the Lecture and Notion sessions, we observed no significant correlation with outcomes. For those who intended to employ the LEARN model or even the hybrid education model, attention could be shifted to emphasize more effective components of the model.

Our study findings that student involvement in English-video, Real-case, and Notion sessions improved their clerkship performance coincides with previous research (19, 28). Educators should encourage students to dedicate more focus to informative videos, clinical cases, and team-based discussions, given the promising outcomes that greater individual student participation was associated with improved performance.

Furthermore, the chi-square test suggested positive outcomes from watching informative videos from YouTube. According to our questionnaire, active participation was defined as listening to the content carefully and summarizing the slide independently. In line with our qualitative analysis, students displaying high-level participation in English-videos were more inclined to outperform their counterparts who participated less in the session in terms of mastery of practical clinical skills (history-taking, physical examination, film reading, and clinical reasoning). Interestingly, several former studies proved the value of educational videos in medical education (22, 23, 29). Based on the proven value of informative videos, an emerging challenge would be the incompatibility of the lowest participation rate with the promising outcomes of the English-video session. In the age of hypermedia, the necessity of blending high-quality e-materials into medical education and the task of involving students in novel teaching methods suggests educators should reform current education modalities and embrace a more comprehensive, contemporary vision for teaching.

To the best of our knowledge, this study might be the very first attempt to employ several education methods simultaneously in the field of medical education. This novel modality promises to foster learning of theoretical knowledge, train students for clinical practice, and prepare them for a future internship, verified by both the after-class quiz and questionnaire.

More details to further improve the LEARN model were investigated through additional questionnaires. As previous research indicated, the multimedia demonstration fostered education outcomes among medical students in PowerPoint-based lectures (30). Likewise, the videos played in the class were PowerPoint-like, as every page was presented with a text section of professional vocabulary, freehand-sketching animation, and simultaneous voiceover in English. Respondents revealed that students accepted the multifaceted way of representation—more than one-half of students paid a considerable proportion of attention to the animation, coinciding with our primary intention to promote the memorization and absorption of knowledge with the aid of vivid and intriguing videos (5, 31). Moreover, only videos in English were employed in class to train students’ reading and listening capacity with professional vocabulary. The inspiring results that near 50% of students concentrated on the voiceover and vocabulary alleviated our concern that teaching professional content entirely in English was beyond the participating students’ level. For those devoted to making progress in medical education, especially for universities in China, despite setting significantly higher requirements for medical students, exposure to international education resources could be a good change to implement.

Given the indispensable role of the advisor in this highly interactive class, this study explored students’ reactions to having an advisor available for discussion and feedback (32). It is encouraging to see that most of the students were content with and encouraged by the interpersonal feedback they received. Considering the relatively small number of respondents and limitation of information, we were not able to determine why two students declined to respond to the Advisor component of our questionnaire. However, the previous study indicated that opinions on mentors were correlated with gender, age, stage of study and teachers’ expression ability (32, 33).

In comparison with the traditional PowerPoint-based lecture, the new LEARN model necessitated implementation of multimedia-based medicine learning materials. Despite a fantastic universe of educational videos from the Internet, almost every student that pursues self-directed online learning can be confronted with multiple high quality resources (34). Therefore, the role of advisors in the LEARN model far surpassed simply being director of class by actively providing appropriate learning materials for students, inevitably shouldering a greater burden than simply teaching in a lecture-based format (35). The premise of LEARN, which requires well-trained teachers, was critical for the broader application of this novel education model. We suggest that teachers who have significant clinical duties may need to reduce their clinical workload to be effective educators.

Our study performed on the LEARN model was limited in some aspects. The design of a cross-sectional study without control inevitably undermines the reliability of our findings. Results only included students’ feedback, lacking assessment to evaluate the quality of this model. There are psychometrically reliable instruments for higher education that have been successfully implemented in medical education, for example Students’ Evaluation of Educational Quality (SEEQ), which includes a string of subscales, including learning value, organization, group interaction, breadth of coverage, assignments, and so forth. We would have employed the scale in our research, however, students enrolled in this program has already graduated from the university, which makes the follow-up investigation unfeasible. We were unable to study long-term retention of knowledge or performance among medical students taught using the LEARN model. It is also unclear whether English learning materials are as readily available for other medical specialties as they are in orthopaedics. As the LEARN model was only tested in the field of orthopaedics for 2–3 months, there will be a lot of work to do before it is proved feasible and useful for medical education in China.

Future research is anticipated to prove the outcomes of the LEARN model. Studies with larger groups of participants and a more diverse range of subjects could explore the broad application of the LEARN model. Moreover, promoting the utilization of the LEARN model in medical schools in China would be a threshold to overcome to put this research into practice.

## Conclusion

This research study substantiated that the LEARN model promotes academic and practical performance in a medical school clerkship. The highly interactive, integrated education modality was rated positively by students and received an excellent participation rate in every session. Besides in Orthopaedic Surgery, an extensive range of clerkships may employ the LEARN model effectively, considering there are common expectations in the requisite abilities of medical students, such as history-taking, physical examination, and clinical reasoning skills. More importantly, the LEARN model may be readily feasible for other medical schools in China or even schools in other countries, given that few resources are required for implementation. Before the application in a broader range, we expect more trials to corroborate its efficacy and to help refine an improved model. For instance, given the positive outcomes of the English-video session as suggested by the qualitative and quantitative analysis, an initial area of refinement can be how to elevate the participation rate of the English-video session, which was the lowest among the five components of the model.

## Data Availability

The raw data supporting the conclusions of this article will be made available by the authors, without undue reservation.
