# Supplementary material for: “LEARN”, a novel teaching method for Chinese clinical clerkship: A cross-sectional study
Source: Front Surg. 2023 Feb 13;10:1113267. doi: 10.3389/fsurg.2023.1113267 (PMC9968847; doi:10.3389/fsurg.2023.1113267)

# 下肢骨折、关节损伤

# Fracture and Joint Injury of Lower Extremity

（见 习 带 教）

(clinical clerkship)

何金深 主治医师

中南大学湘雅三医院 骨科

Jinshen He

Department of Orthopedic Surgery

Third Xiangya Hospital of Central South University

# 课程安排 class arrangement

- 知识回顾 Lecture-based learning — 60min
- 观看视频、回答问题 English-video quizzes — 20min
- 阅片、问诊、体查演练 Advisor-guided training — 30min
- 临床病例学习 Real-case practice — 60min
- 病例诊断与治疗方案 Notions discussed by teams — 60min

# 知识回顾 Lecture-based learning

- 相关定义
  Definitions
- 下肢骨折的诊断
  Diagnosis of fracture of lower extremity
- 骨科阅片要点
  Key points of radiography
- 治疗原则
  Treatment principles

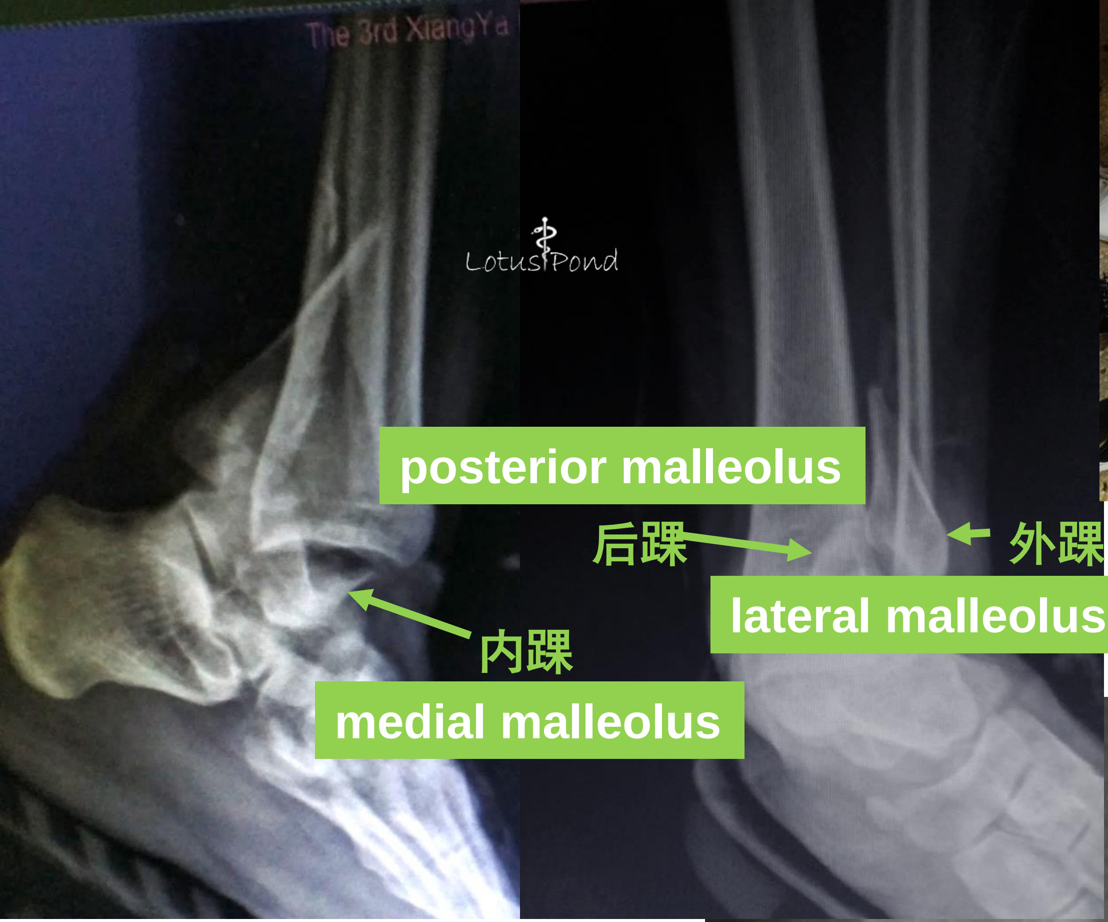


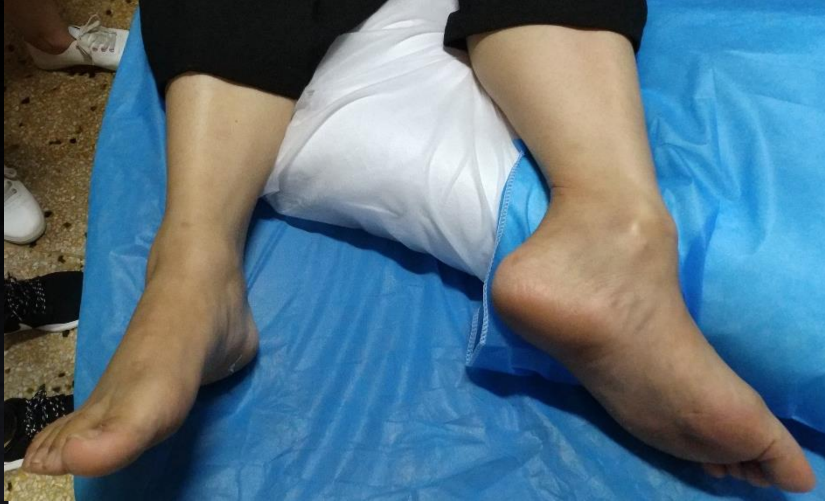

16岁女性，扭伤致左踝疼痛4小时

16, f, 4h pain of left ankle by sprain

1. 识骨寻踪，诊断是？
   Diagnosis？
2. 骨折定义？
   Definition of fracture？
3. 脱位定义？
   Definition of dislocation？
4. 骨折治疗原则？
   Treatment principle?

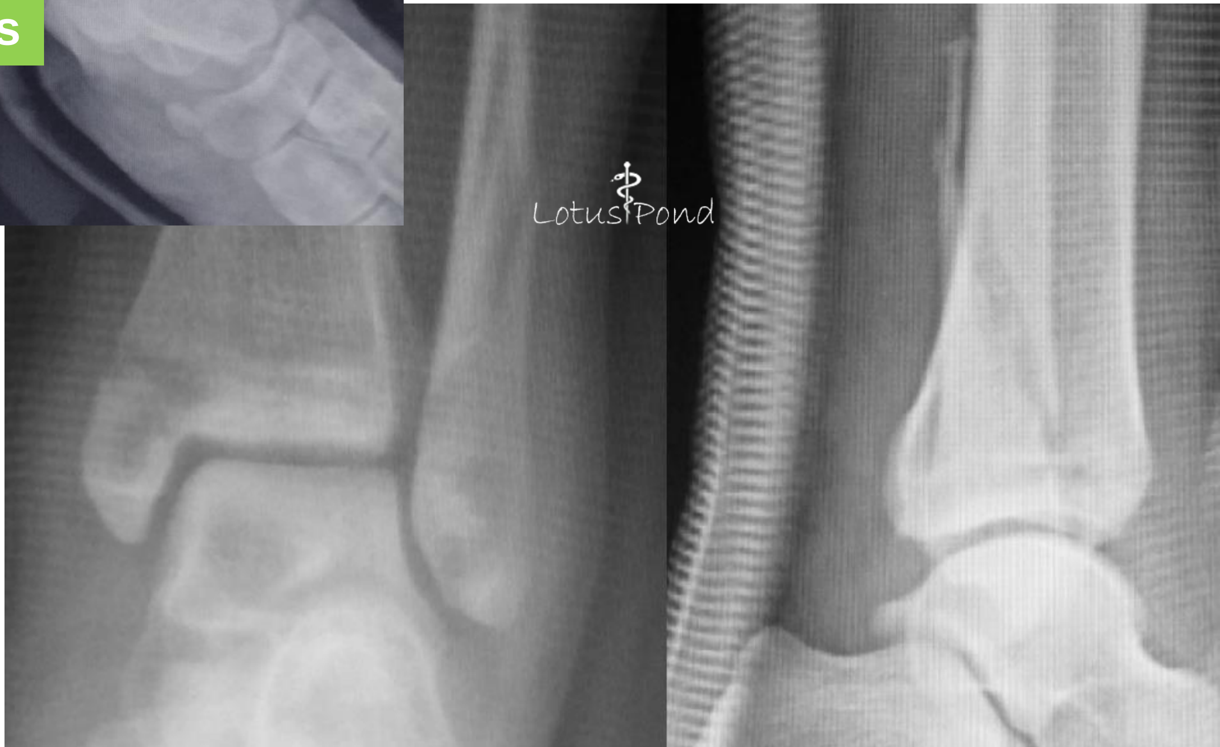

# 课程安排 class arrangement

- 知识回顾 Lecture-based learning 60min
- 观看视频、回答问题 English-video quizzes 20min
- 阅片、问诊、体查演练 Advisor-guided training 30min
- 临床病例学习 Real-case practice 60min
- 病例诊断与治疗方案 Notions discussed by teams 60min

OSMOSIS.org

OSMOSIS.org
2022 Edition

# 课程安排 class arrangement

- 知识回顾 Lecture-based learning — 60min
- 观看视频、回答问题 English-video quizzes — 20min
- 阅片、问诊、体查演练 Advisor-guided training — 30min
- 临床病例学习 Real-case practice — 60min
- 病例诊断与治疗方案 Notions discussed by teams — 60min

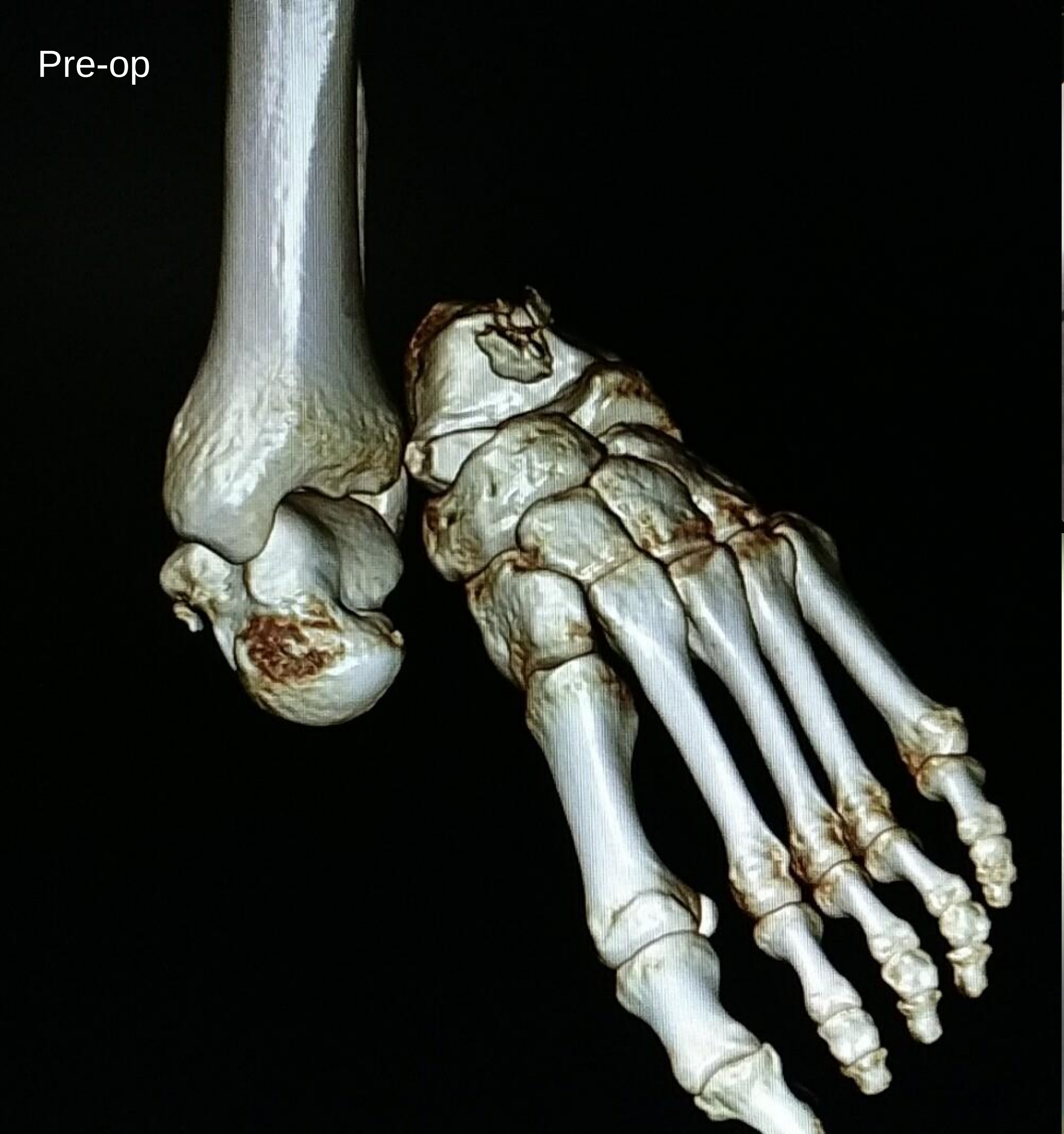
Pre-op

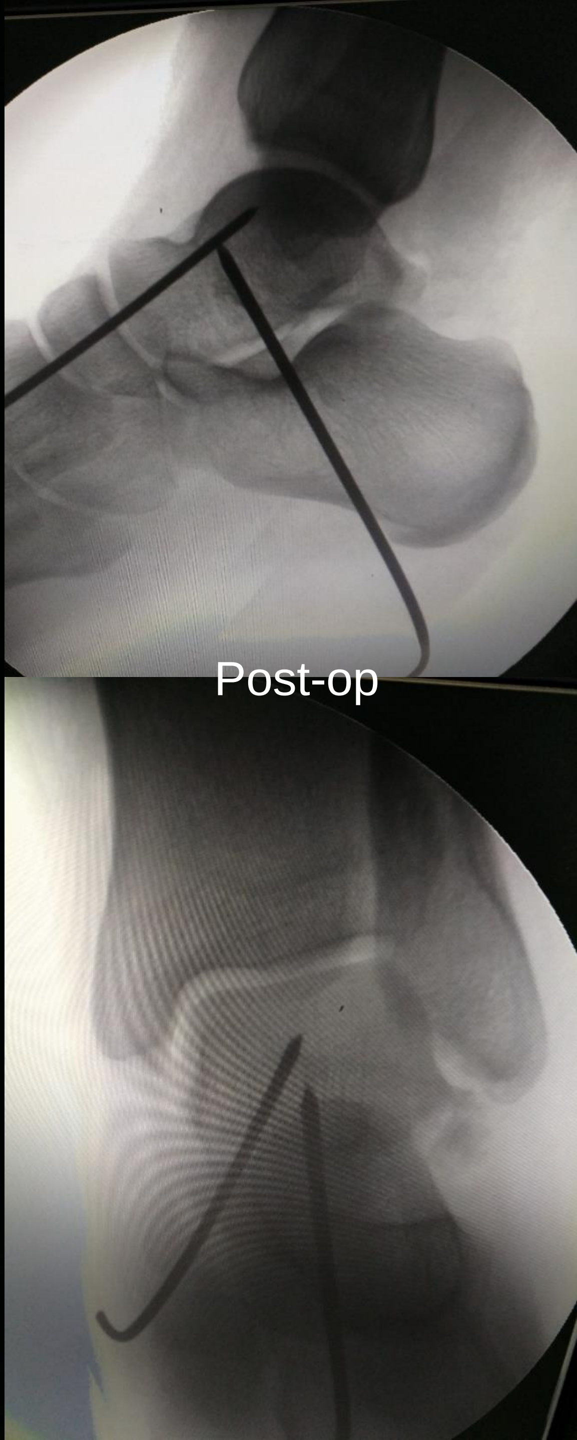
Post-op

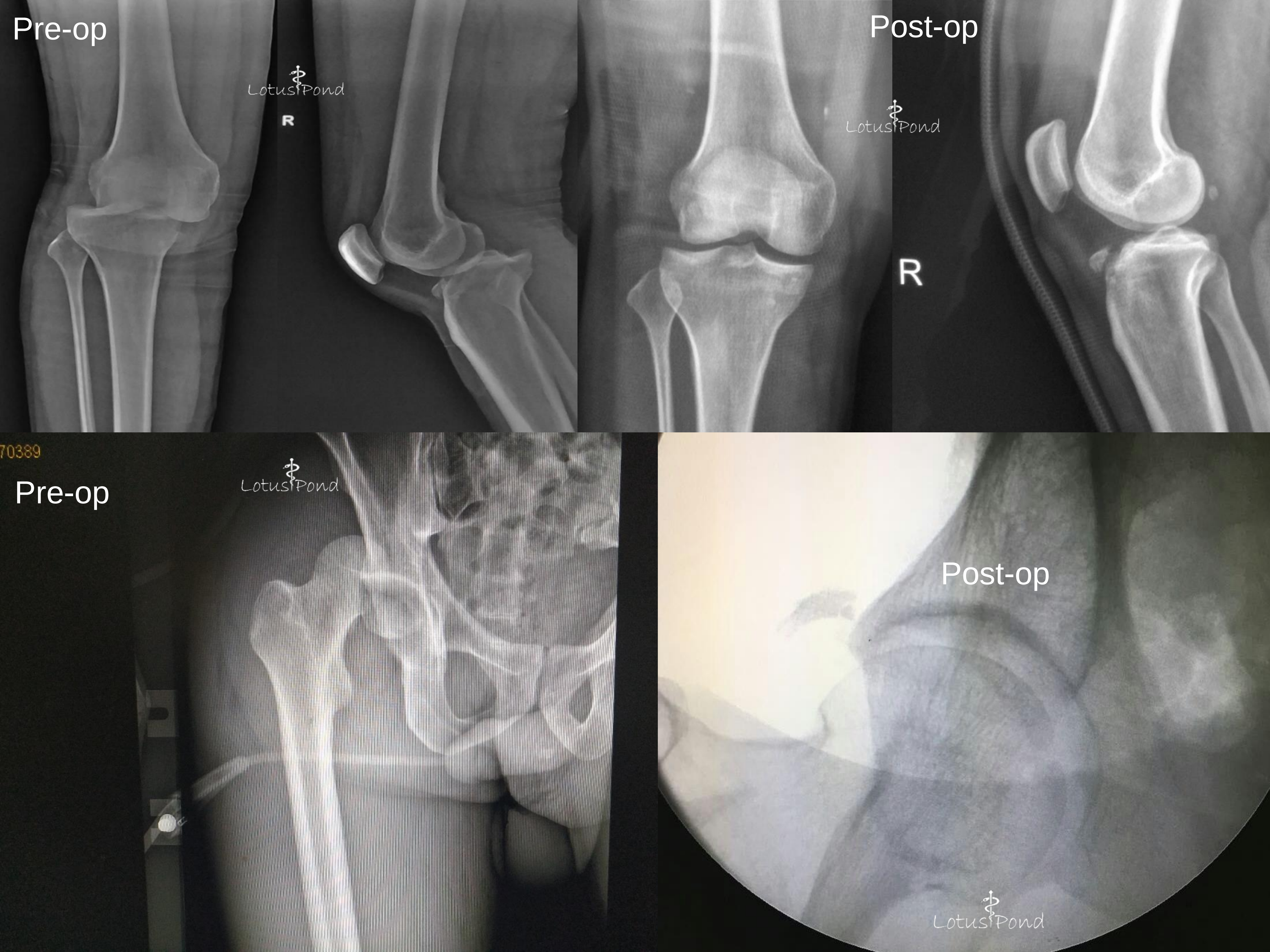
Pre-op
LotusPond
R
Post-op
LotusPond
R
70389
Pre-op
LotusPond
Post-op
LotusPond

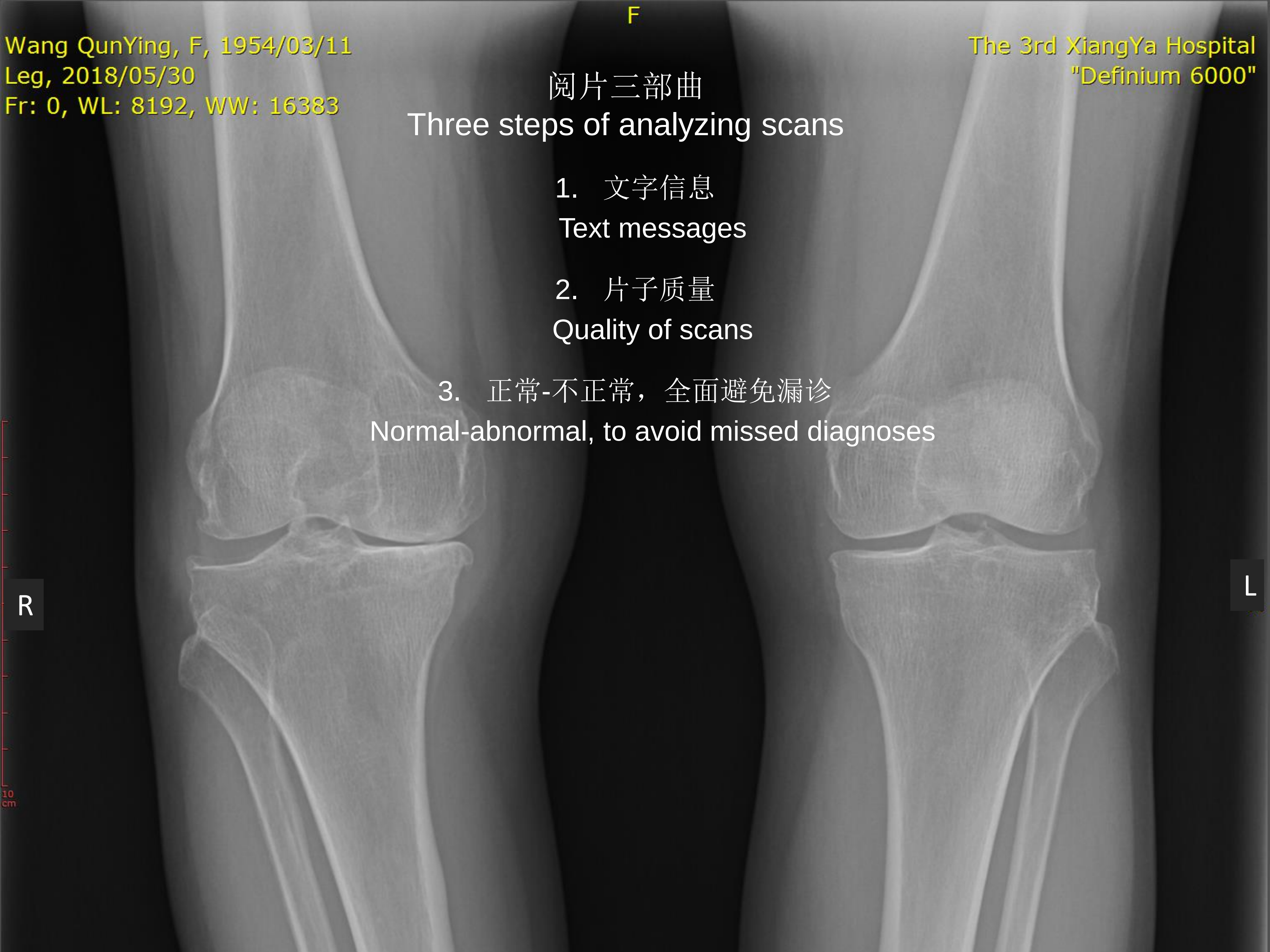


# 阅片三部曲
# Three steps of analyzing scans

1. 文字信息
   Text messages
2. 片子质量
   Quality of scans
3. 正常-不正常，全面避免漏诊
   Normal-abnormal, to avoid missed diagnoses

# 课程安排 class arrangement

- 知识回顾 Lecture-based learning — 60min
- 观看视频、回答问题 English-video quizzes — 20min
- 阅片、问诊、体查演练 Advisor-guided training — 30min
- 临床病例学习 Real-case practice — 60min
- 病例诊断与治疗方案 Notions discussed by teams — 60min

- 主诉 （xx致左x疼痛4h）

  **Chief complaint：4h pain caused by xxx**

- 现病史 **history of present illness**

  （外伤及机制？）——意外伤害 **or** 坠落

**(injury or other mechanisms) —accident injury or falling injury**

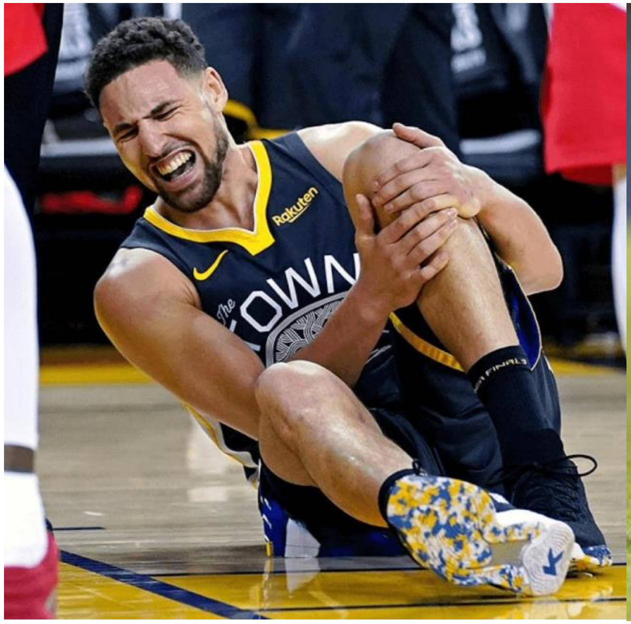

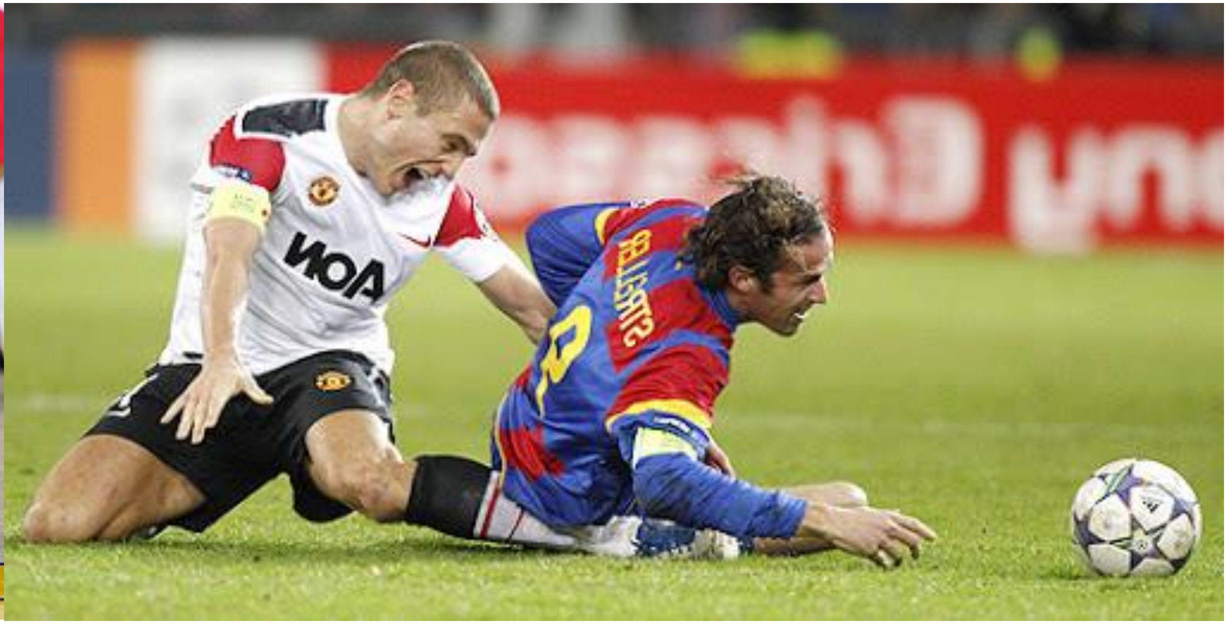

- 主诉 （xx致左x疼痛4h）
  Chief complaint：4h pain caused by xxx
- 现病史 history of present illness
  （外伤及机制？）——意外伤害 or 坠落
  (injury or other mechanisms) —accident injury or falling injury

**（疼痛性质时间、伴随症状、加重缓解因素）**

**(pain's characters, lasting period, accompanying symptoms, aggravating and mitigating factors)**

……

- 主诉 （xx致左x疼痛4h）
  Chief complaint：4h pain caused by xxx
- 现病史 history of present illness
  （外伤及机制？）——意外伤害 or 坠落
  (injury or other mechanisms) —accident injury or falling injury
  （疼痛性质时间、伴随症状、加重缓解因素）
  (pain’s characters, lasting period, accompanying symptoms, aggravating and mitigating factors)

……

- 既往史、个人史、婚育史、家族史
  Past history, personal history, obstetric history, family history
- 过敏allergy 月经及备孕 Menstrual history or pregnancy

# 课程安排 class arrangement

- 知识回顾 60min
  Lecture-based learning
- 观看视频、回答问题 20min
  English-video quizzes
- 阅片、问诊、体查演练 30min
  Advisor-guided training
- 临床病例学习 60min
  Real-case practice
- 病例诊断与治疗方案 60min
  Notions discussed by teams

# 骨科体格检查原则
# Physical examination principle

- ✓ 充分暴露、双侧对比
  Appropriately expose body, bilaterally compare
- ✓ 先健侧后患侧，先主动后被动
  Normal side and active action first
- ✓ 全面、反复、轻柔、到位
  Comprehensive, reduplicative, gentle, precise
- ✓ 视、触、动、量、特殊检查、肌力感觉
  Inspection, palpation, movement, measure, specific examination, power and sensation

# 望　　诊

# Inspection

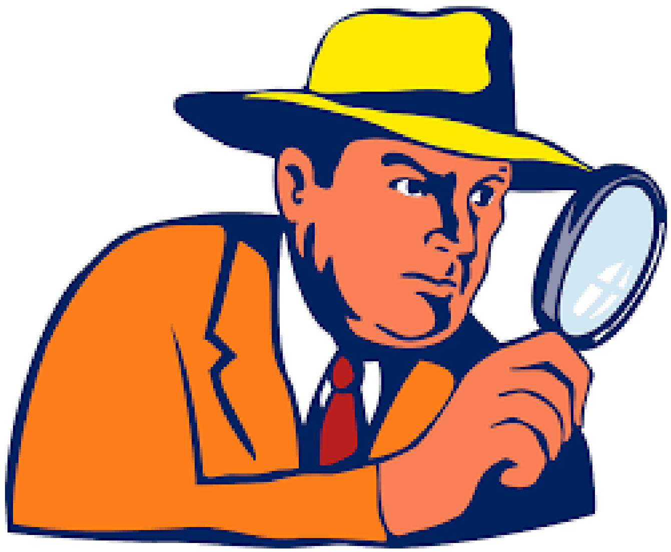

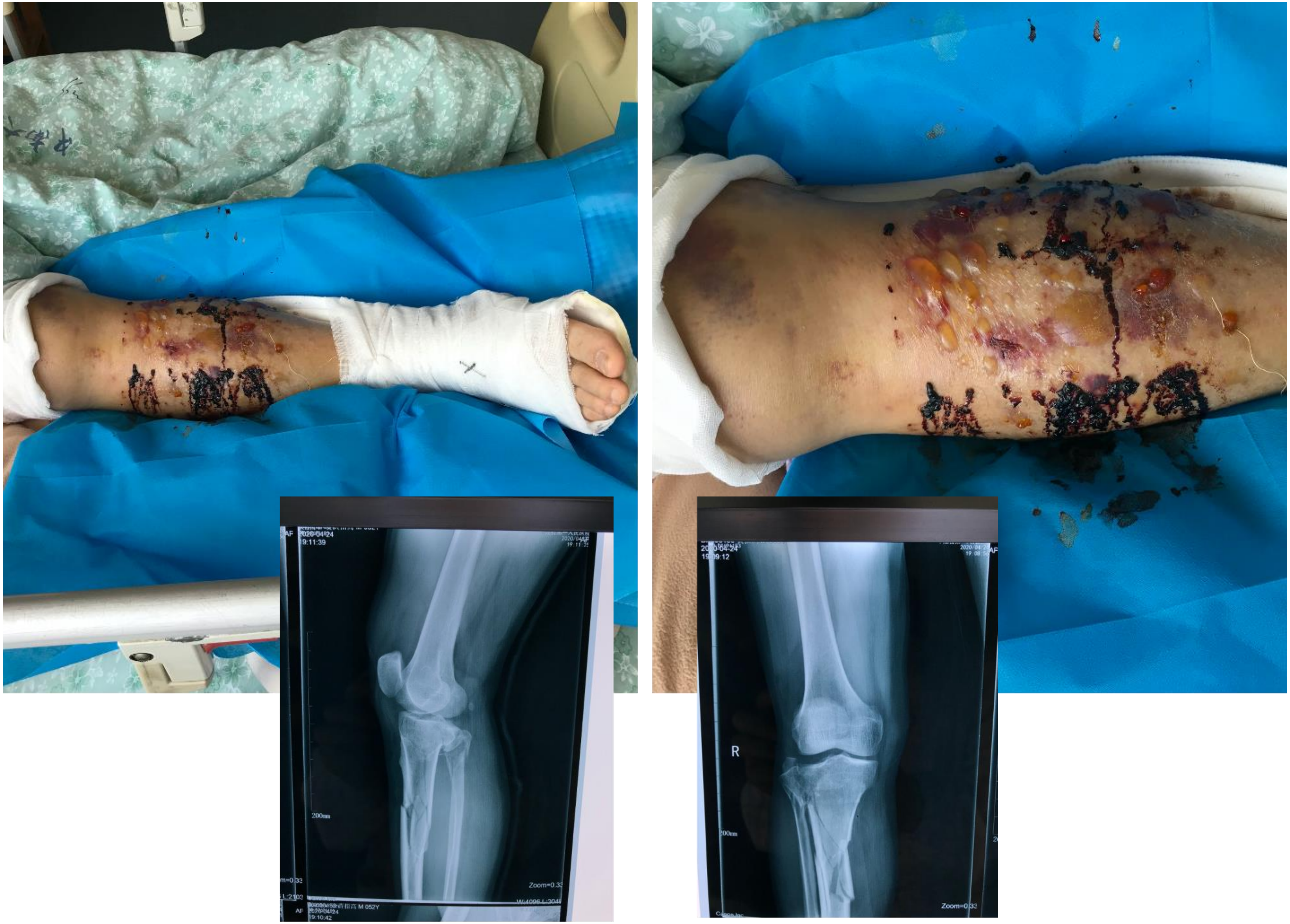
2020/04/24
19:11:39
200mm
R

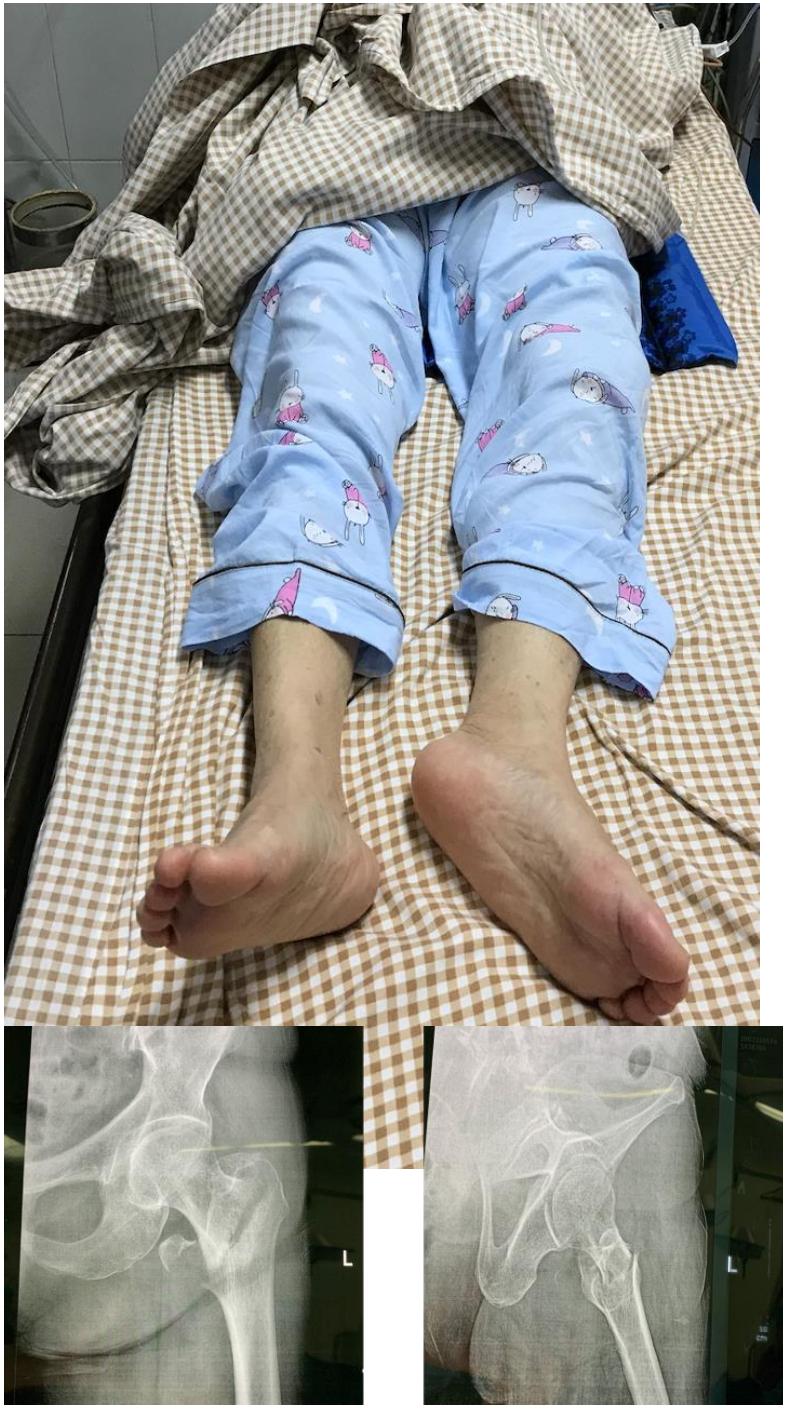
L
L
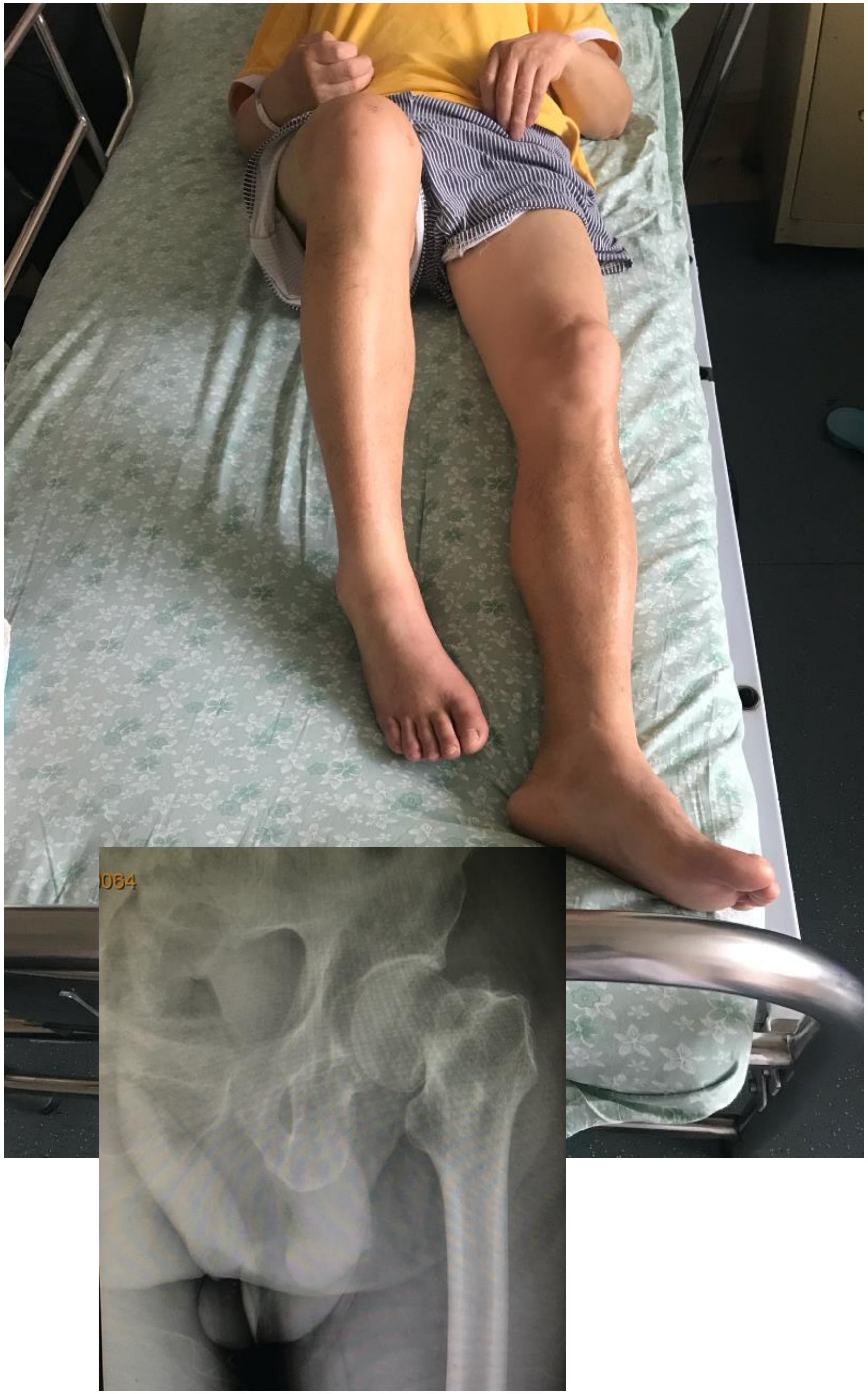
064

# 触　　诊

# Palpation

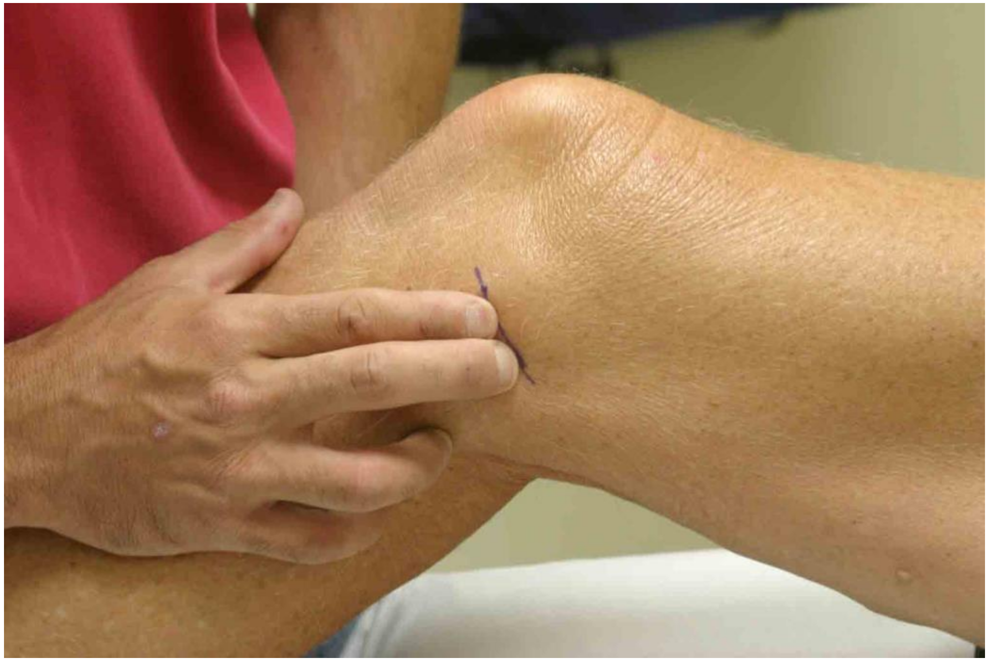

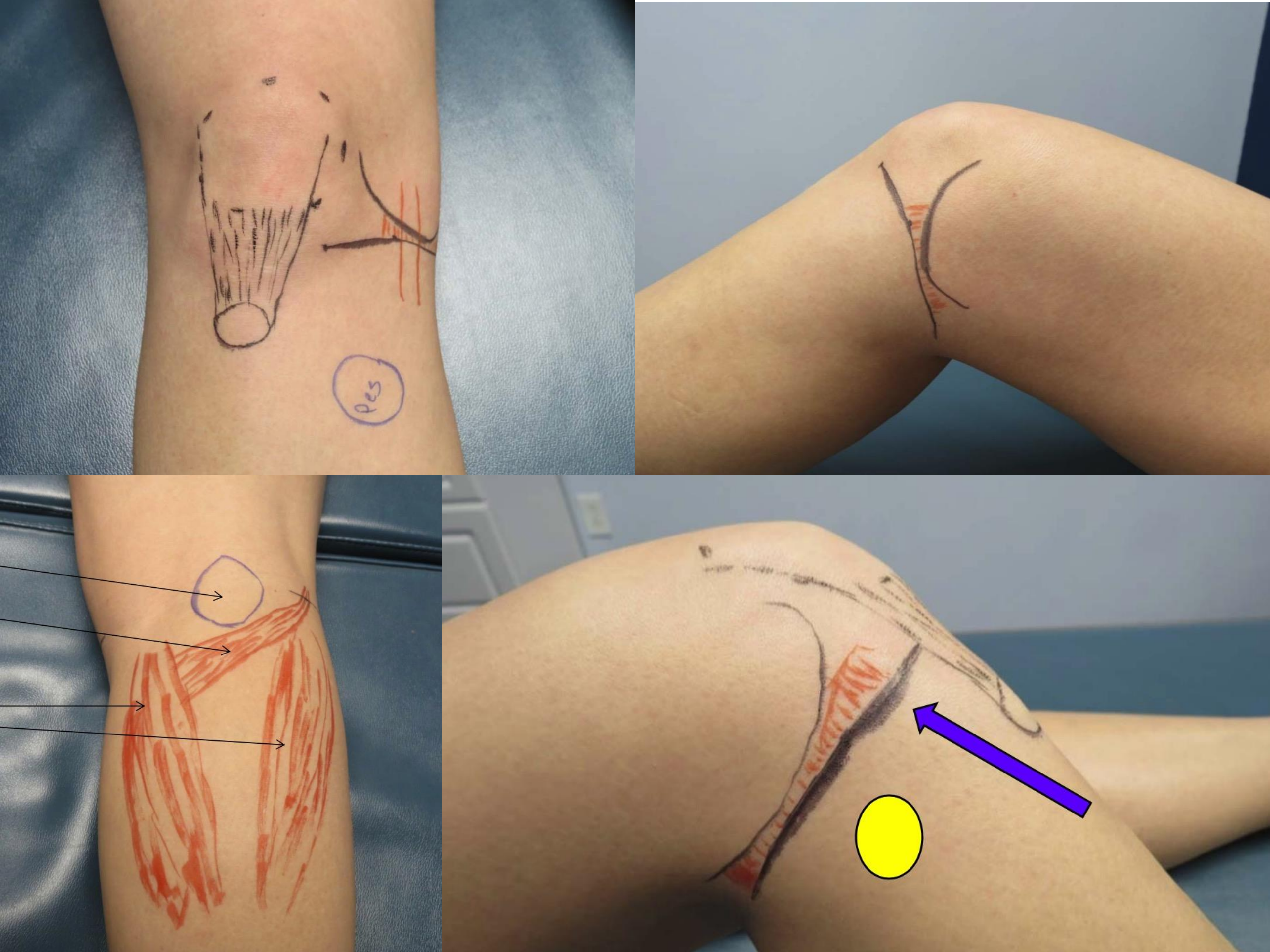
Pes

# 活动度检查ROM

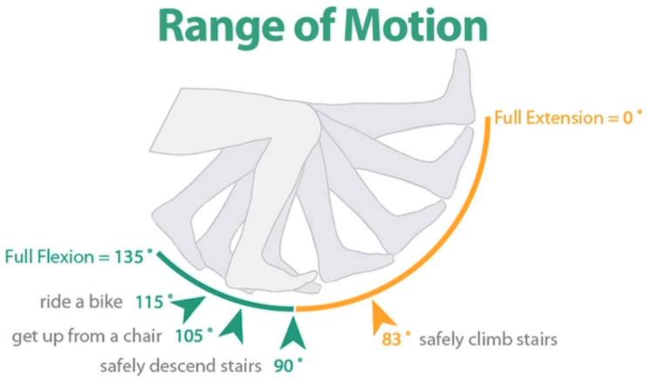
Range of Motion
Full Extension = 0°
Full Flexion = 135°
ride a bike 115°
get up from a chair 105°
safely descend stairs 90°
83° safely climb stairs

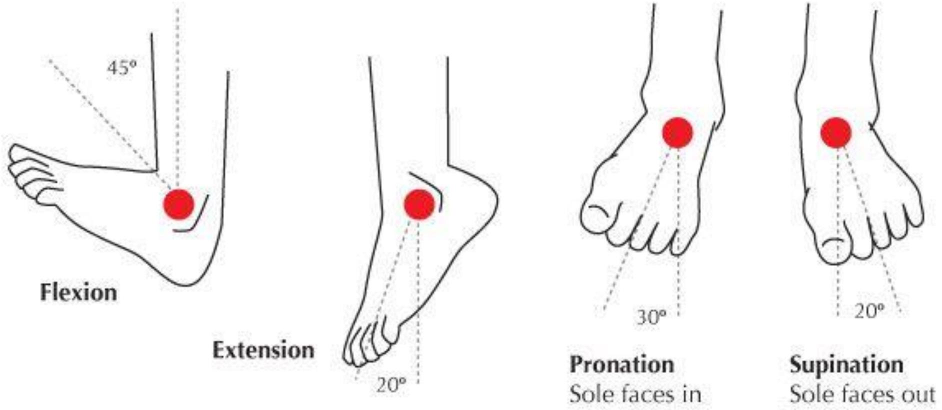
45°
Flexion
Extension
20°
30°
Pronation
Sole faces in
20°
Supination
Sole faces out

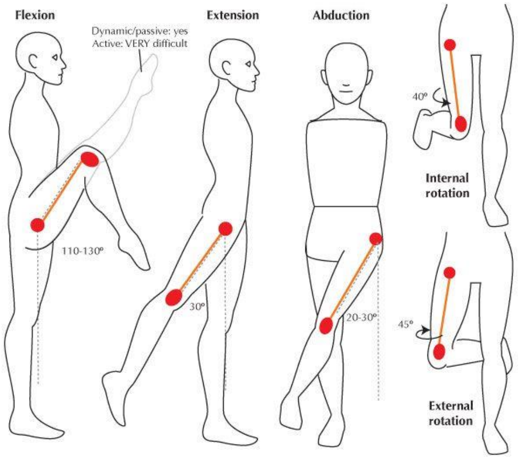
Flexion
Dynamic/passive: yes
Active: VERY difficult
110-130°
Extension
30°
Abduction
20-30°
40°
Internal rotation
45°
External rotation

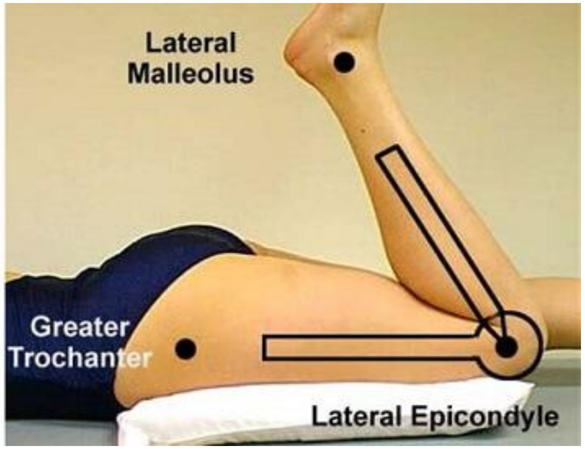
Lateral Malleolus
Greater Trochanter
Lateral Epicondyle

# 量measure

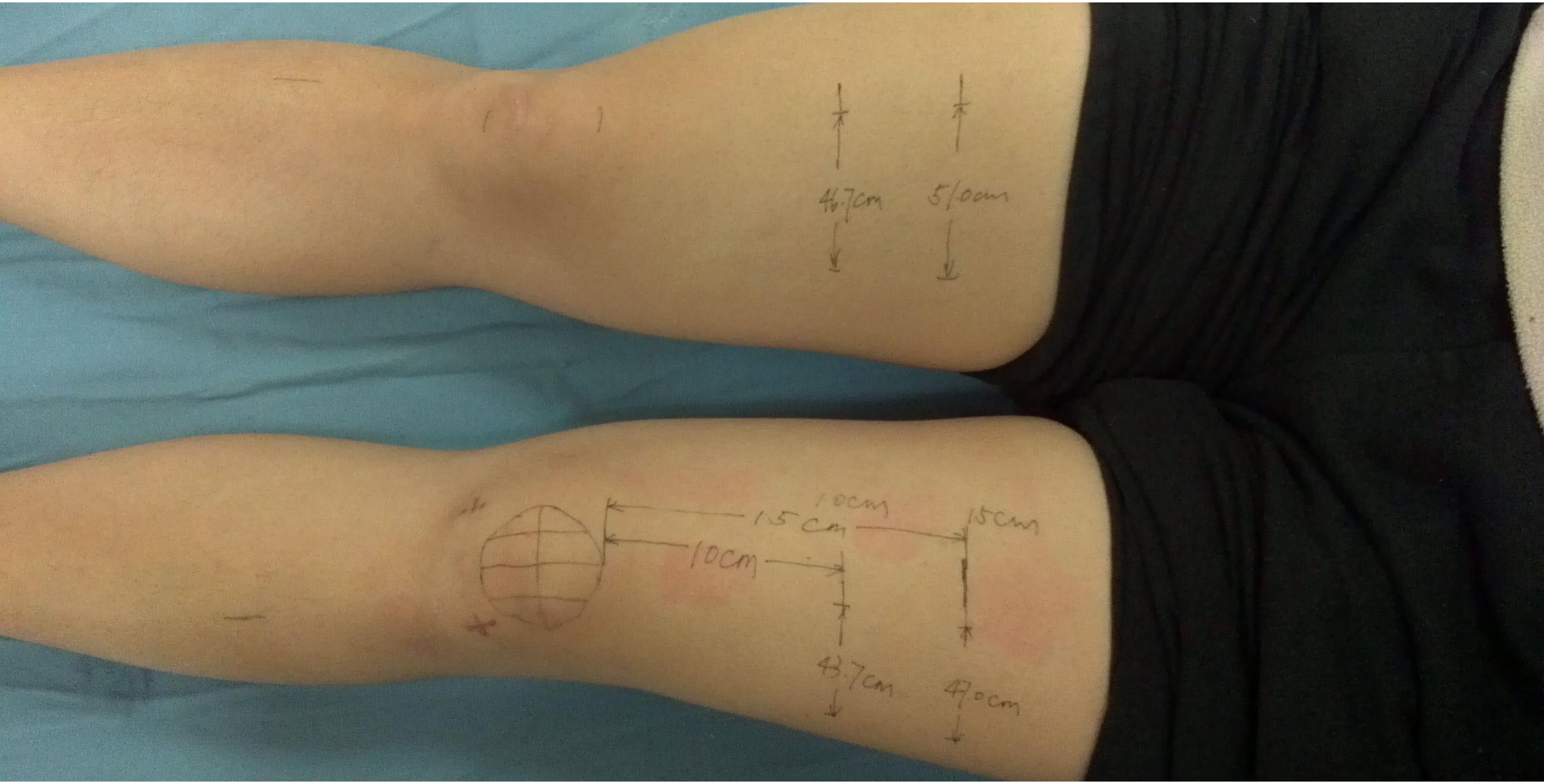

# 特殊检查

# Specific examination

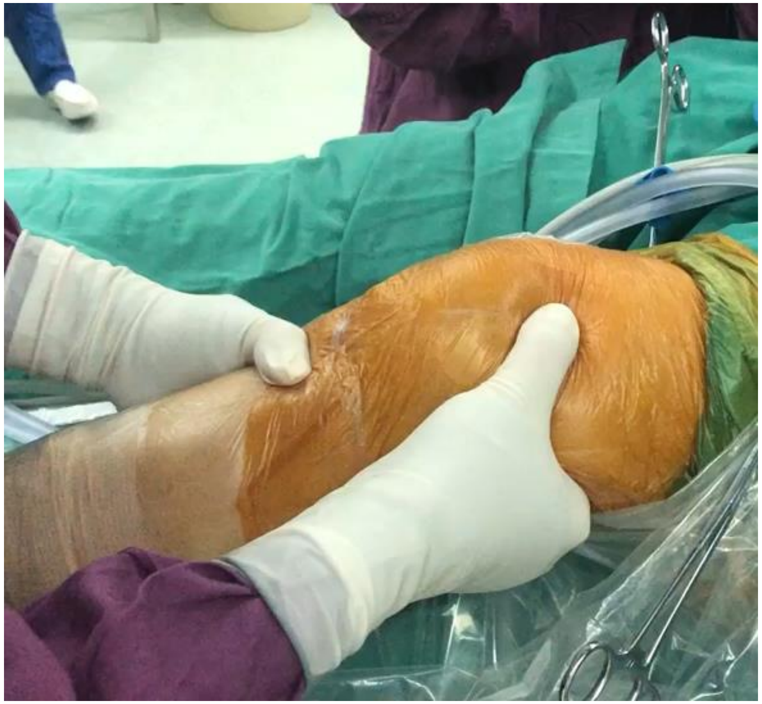

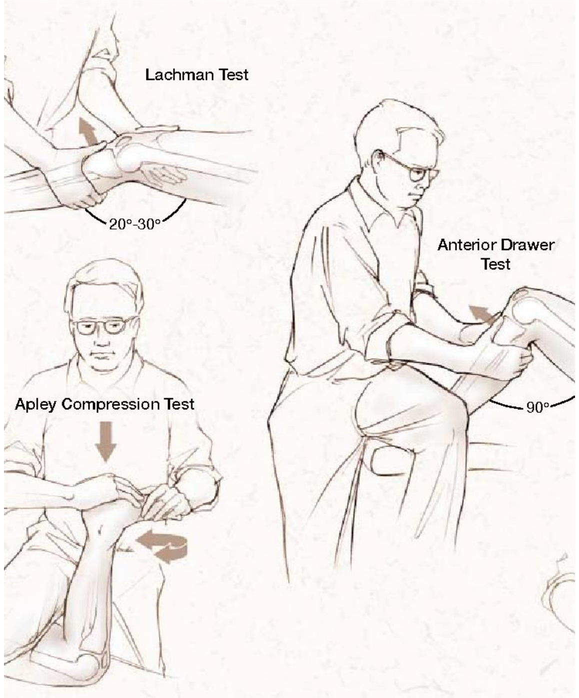


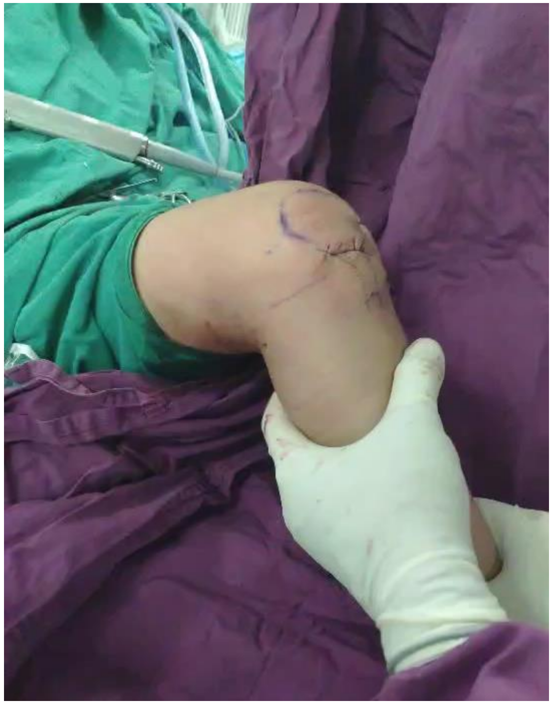

# 课程安排 class arrangement

- 知识回顾 Lecture-based learning — 60min
- 观看视频、回答问题 English-video quizzes — 20min
- 阅片、问诊、体查演练 Advisor-guided training — 30min
- 临床病例学习 Real-case practice — 60min
- 病例诊断与治疗方案 Notions discussed by teams — 60min

# 课程安排 class arrangement

- 知识回顾 60min
  Lecture-based learning
- 观看视频、回答问题 20min
  English-video quizzes
- 阅片、问诊、体查演练 30min
  Advisor-guided training
- 临床病例学习 60min
  Real-case practice
- 病例诊断与治疗方案 60min
  Notions discussed by teams

# 病例汇报及讲解
## Case report and discussion

3小组（每组5-6人）

3 groups (per 5-6 students)

每组汇报时间：6min

Report time: 6min

相互补充提问：4min

Intergroup questions: 4min

老师补充及提问：10min

Questions from teacher: 10min

中南大学湘雅三医院 外科教研室
The Third Xiangya Hospital of Central South University

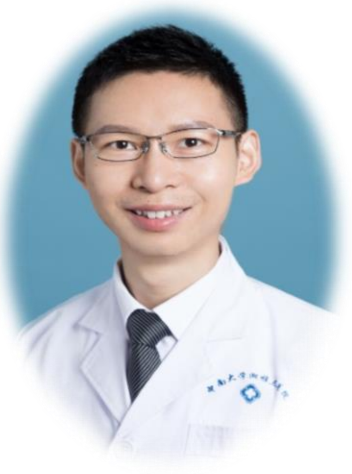

# 谢谢您的聆听！

**Thanks for listening！**

部分图片和视频来自Youtube和Google，仅做教学用

Several pictures and videos are from Youtube or Google, which are only used only for teaching.

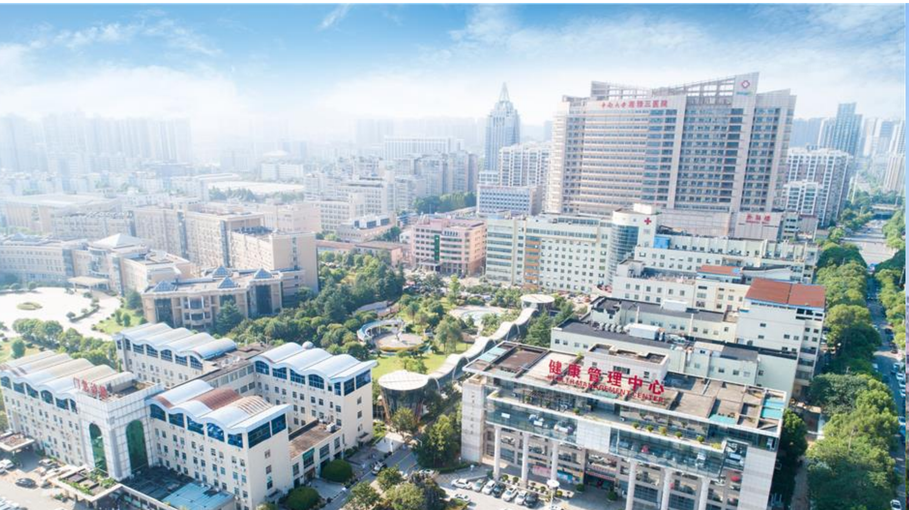


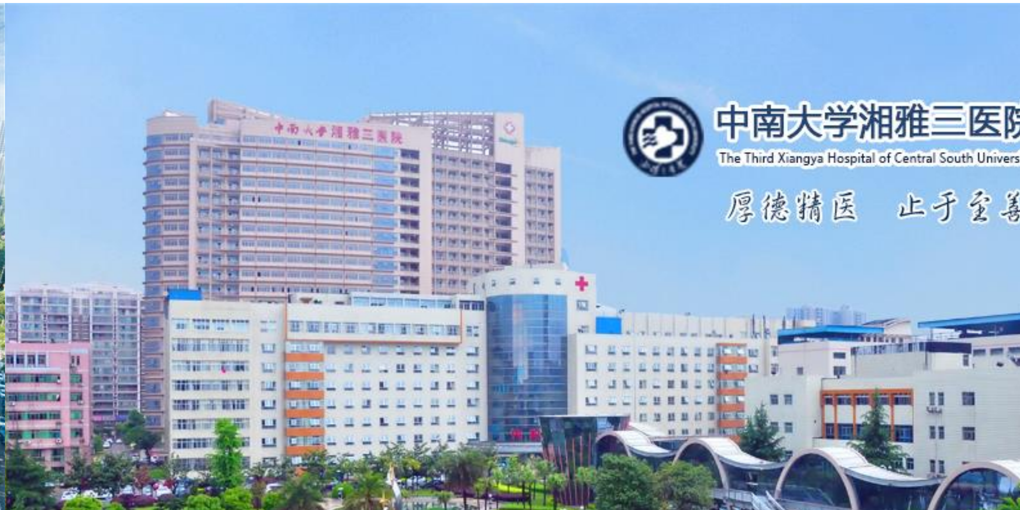

Supplement: Supplementary file 1 [file Presentation1.pdf]
