# Supplementary material for: “LEARN”, a novel teaching method for Chinese clinical clerkship: A cross-sectional study
Source: Front Surg. 2023 Feb 13;10:1113267. doi: 10.3389/fsurg.2023.1113267 (PMC9968847; doi:10.3389/fsurg.2023.1113267)
Supplement: Supplementary file 2 [file Datasheet1.docx]

| Table S1 What students focused on during English-video | | |
| --- | --- | --- |
|  | number of students | percent |
| Animation | 50 | 51.02% |
| Voiceover | 18 | 18.37% |
| Vocabulary | 30 | 30.61% |

| Table S2 Students' willingness to be SP | | |
| --- | --- | --- |
|  | number of students | percent |
| Selected as SP | 15 | 15.31% |
| Not SP but willing to be | 70 | 71.43% |
| Unwilling to be SP | 6 | 6.12% |
| Not to answer | 7 | 7.14% |

| Table S3 Students' opinions on the surgery video | | |
| --- | --- | --- |
|  | number of students | percent |
| It's essential | 89 | 90.82% |
| Not to answer | 9 | 9.18% |

| Table S4 The adequate number of questions | | |
| --- | --- | --- |
|  | number of students | percent |
| ≥5 | 5 | 5.10% |
| 4 | 2 | 2.04% |
| 3 | 26 | 26.53% |
| 2 | 53 | 54.08% |
| 1 | 12 | 12.24% |

**Supplementary figure 1**


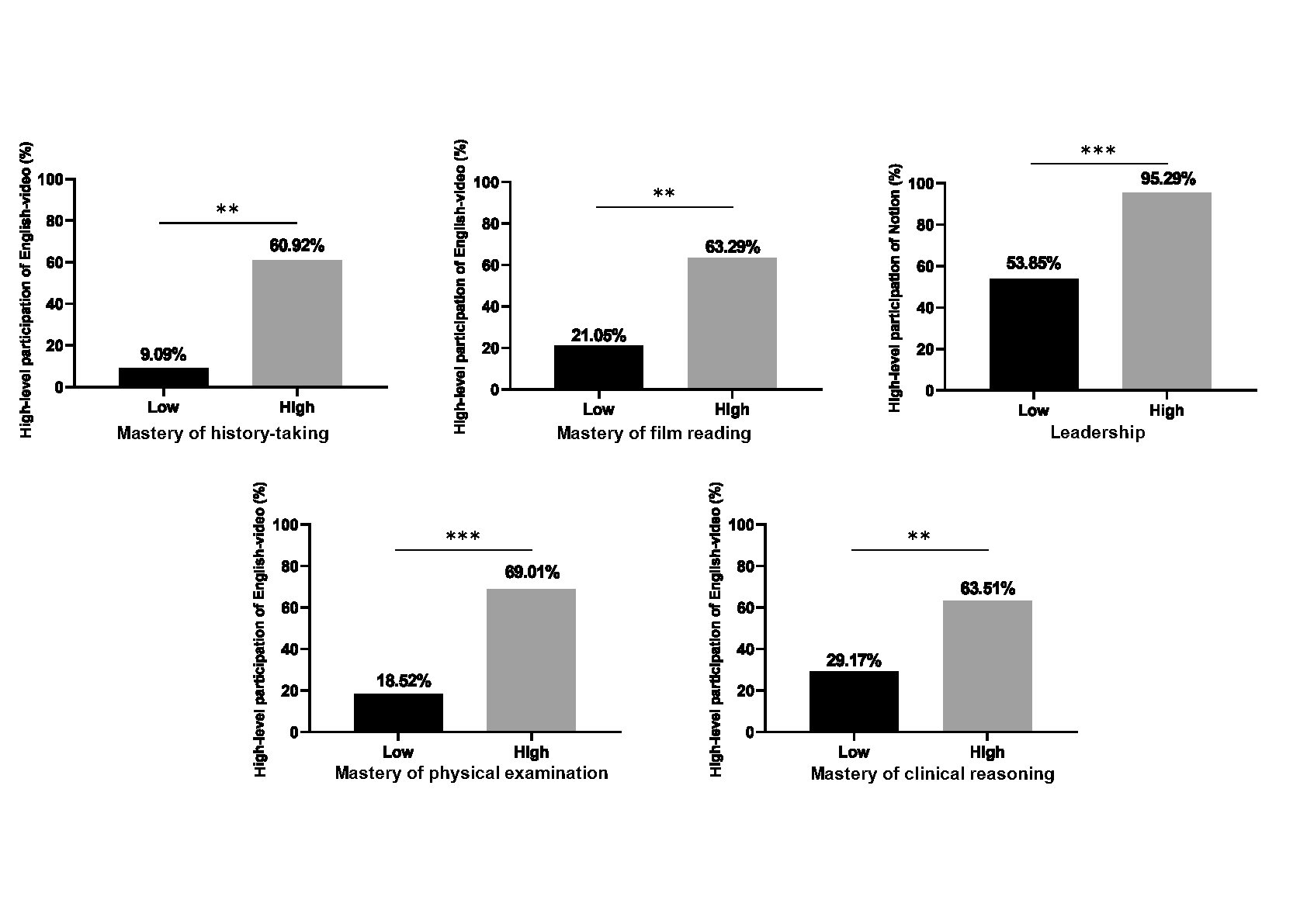


**Additional File 1 Questionnaire**

- Individual information (Anonymous)
- Age
- Gender
- The last four numbers of student ID
- Your study group number
- Rating of the lecture given by the teacher (J. He) (5-Likert scale)

1. Passion in class
2. Interaction with students
3. Overall performance in teaching

- Rating of English-video section (5-Likert scale)

1. Being helpful for reviewing
2. Assisting vocabulary learning
3. Interest added by English animation
4. Enhancing participation of class via quiz

- What were you focusing on during the English-video?

1. Guessing the English words in the animation
2. Listening to the voiceover and trying to comprehend
3. Trying to conclude the context from the animation

- Participation of English-video (5-point scale)

1. I listened to and comprehended the video even if it was not my turn to answer questions.
2. I actively helped classmates answer questions.
3. I translated the video context based on Chinese textbook

- Participation of clinical skill training (3-point scale)

1. Favorable
2. Unfavorable
3. Not to answer

- Rating of clinical skill training (5-point scale)

1. Being helpful for knowledge integration
2. Assisting sequent practice on patients
3. Enhancing the understanding of orthopedics

- Are you willing to be standardized patients in class for knee-joint examination?

1. Selected as SP
2. Not SP but willing to be
3. Unwilling to be SP
4. Not to answer

- Rating of notion exchange during the case report (5-point scale)

1. Fostering comprehension of knowledge
2. Strengthening ability of clinical reasoning
3. Enlightening passion for future internship
4. Leaving deep impression

- Participation of bedside practice (5-point scale)

1. I joined in the clerkship fully.
2. I carefully listened to the case reports from other groups.
3. I could lead team members to conclude the case.
4. I understood and absorbed teacher’s comments on the case report.

- Will you actively seek for solutions of diagnosis, antidiastole and treatment by yourself?

1. Yes
2. No

- Is it necessary to watch the surgery video at the end of class?

1. Yes
2. No
3. Not to answer

- Self-rating of confidence upon following aspects (5-point scale):

1. Mastery of basic theoretical knowledge of orthopedics
2. Mastery of inquiring
3. Mastery of physical examination
4. Mastery of film reading
5. Mastery of clinical reasoning (diagnosing and proposing for further treatment)

- The adequate amount of questions in class.

1. 0
2. 1
3. 2
4. 3
5. 4
6. ≥5

- How do you feel about the feedback from the teacher?

1. Helpful and inspiring
2. Neutral
3. Frustrating and harmful
